# Growth‐dependent heterogeneity in the DNA damage response in *Escherichia coli*

**DOI:** 10.15252/msb.202110441

**Published:** 2022-05-27

**Authors:** Sebastián Jaramillo‐Riveri, James Broughton, Alexander McVey, Teuta Pilizota, Matthew Scott, Meriem El Karoui

**Affiliations:** ^1^ Institute of Cell Biology and SynthSys University of Edinburgh Edinburgh UK; ^2^ Department of Applied Mathematics University of Waterloo Waterloo ON Canada; ^3^ Present address: OGI Bio Ltd Edinburgh UK

**Keywords:** bacterial physiology, DNA repair, single‐cell, DNA Replication, Recombination & Repair, Microbiology, Virology & Host Pathogen Interaction

## Abstract

In natural environments, bacteria are frequently exposed to sub‐lethal levels of DNA damage, which leads to the induction of a stress response (the SOS response in *Escherichia coli*). Natural environments also vary in nutrient availability, resulting in distinct physiological changes in bacteria, which may have direct implications on their capacity to repair their chromosomes. Here, we evaluated the impact of varying the nutrient availability on the expression of the SOS response induced by chronic sub‐lethal DNA damage in *E. coli*. We found heterogeneous expression of the SOS regulon at the single‐cell level in all growth conditions. Surprisingly, we observed a larger fraction of high SOS‐induced cells in slow growth as compared with fast growth, despite a higher rate of SOS induction in fast growth. The result can be explained by the dynamic balance between the rate of SOS induction and the division rates of cells exposed to DNA damage. Taken together, our data illustrate how cell division and physiology come together to produce growth‐dependent heterogeneity in the DNA damage response.

## Graphical abstract

Bacteria experience various levels of nutrient availability and are often exposed to DNA damage. The DNA damage response (SOS response) is analyzed in *Escherichia coli* at the single‐cell level in several nutrient conditions.

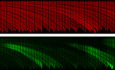

## Introduction

Bacteria are remarkable in their capacity to respond favourably to different environmental conditions, including variations in nutrient availability and perturbations from many different stresses such as oxidative damage or temperature changes. Natural environments vary in their levels of nutrients, affecting the growth of micro‐organisms. For example, *Escherichia coli* has been estimated to divide every 3 h inside the intestine, whereas estimates for division time in the urine (bladder) are about 20–30 min (Myhrvold *et al*, 2015; Forsyth *et al*, 2018). These variations in growth rate can have important consequences for bacterial stress response because they impose constraints on the capacity of bacteria to modify their proteomes (Hui *et al*, 2015). This is particularly true for stresses induced by exposure to antibiotics, as the targets of most antibiotics are growth‐related processes (Lewis, 2013) and variations in growth rate correlate with molecular and physiological changes in bacteria (Bremer & Dennis, 2008). For example, the analysis of the interplay between growth‐related changes and the response to antibiotics has been useful in gaining a quantitative understanding of how bacteria respond to ribosome‐targeting antibiotics (Greulich *et al*, 2015; Pinheiro *et al*, 2021). Yet, the connection between growth‐related changes and the response to other stresses, such as DNA damage, has not been explored.

DNA damage is one of the most ubiquitous types of stress encountered by bacteria. It can arise from external sources such as exposure to UV light or to DNA damaging agents, for example, quinolone antibiotics (Gutierrez *et al*, 2018). Impaired DNA replication leads to the accumulation of DNA Double Strand Breaks (DSBs) at inactivated replication forks, providing a direct link between the cell cycle and DNA damage. Spontaneous DSBs have been linked to stalling of the replisome by obstacles, and/or a replication fork encountering DNA nicks and gaps (Kuzminov, 2001; Michel *et al*, 2004, 2018). DNA replication is also involved in the formation of DSBs after exposure to quinolones (Pohlhaus & Kreuzer, 2005; Drlica *et al*, 2008). DSBs are the most deleterious type of DNA damage as they lead to loss of genetic information. They are repaired by homologous recombination where the broken chromosome is repaired using an intact homologous copy as a template. Homology search is catalysed by RecA which forms a nucleoprotein filament on single‐strand DNA and promotes strand invasion after a homologous copy has been found (Del Val *et al*, 2019). This also leads to the induction of the SOS response (see below).

Changes in growth rates have important consequences on DNA replication in bacteria. In *E. coli*, in rich nutrient conditions, replication of the chromosome is estimated to take about 40 min, and segregation/septation to take another 20 min, for a cell cycle time of approximately 60 min (Bremer & Dennis, 2008). When cells divide faster than 60 min, they initiate several overlapping rounds of DNA replication (a process referred to as “multifork replication”) (Cooper & Helmstetter, 1968; Skarstad & Katayama, 2013). Thus, DNA content and the number of replication forks are higher in fast‐growing cells than in slow‐growing ones. For example, *E. coli* cells doubling in 30 min would contain an average of ~5 replication forks per cell, while this average drops to ~0.36 for cells doubling in 3 h. Although DSBs are likely to arise more frequently in fast‐growth conditions because of the high number of replication forks, it is possible that the presence of multiple partial copies of the chromosome facilitates homology‐dependent repair. This raises the question of how DSB repair may vary with growth conditions. Moreover, growth conditions influence gene expression in bacteria (Hui *et al*, 2015), resulting in less capacity to induce stress‐response genes in fast‐growth conditions. Thus, bacteria may vary in their capacity to induce the DNA damage response depending on the growth conditions.

*Escherichia coli* responds to DNA damage by inducing expression of the SOS regulon (Radman, 1975; Ari, 1985; Erill *et al*, 2007; Kreuzer, 2013), which is important for bacteria to survive DNA damaging conditions (Mount *et al*, 1972; Lin & Little, 1988; Mo *et al*, 2016). The SOS regulon is controlled by the LexA transcriptional repressor, which normally binds to SOS promoters thus limiting their transcription. In DNA damaging conditions, LexA binds to the RecA nucleoprotein filaments, resulting in LexA self‐cleavage, and leading to the expression of SOS genes (Little, 1991; Butala *et al*, 2011; Kovačič *et al*, 2013). In *E*. *coli*, about 30 genes are under the control of LexA (Fernández De Henestrosa *et al*, 2000), including genes involved in DNA repair (e.g. *recA*), inhibition of cell division (*sulA*), translesion DNA synthesis, toxin‐antitoxin modules, and the *lexA* gene itself (Kreuzer, 2013; Baharoglu & Mazel, 2014). In addition to enabling survival under DNA damaging conditions, SOS induction can contribute to an increased rate of mutagenesis (Vaisman *et al*, 2012; Dapa *et al*, 2017; Pribis *et al*, 2019), an increased prevalence of antibiotic tolerance (Dörr *et al*, 2009; Wu *et al*, 2015), and misregulation of the transfer rate of conjugative plasmids and other mobile elements (Beaber *et al*, 2004; Baharoglu *et al*, 2010; Fornelos *et al*, 2016).

Previous reports have shown that SOS expression is heterogeneous in single cells, both in response to DNA damage induced by exogenous agents (Friedman *et al*, 2005; Culyba *et al*, 2018; Uphoff, 2018; Mitosch *et al*, 2019), and when the response is induced by spontaneous DNA damage (Pennington & Rosenberg, 2007; Massoni *et al*, 2012). Heterogeneity in the levels of SOS induction may arise from multiple sources including: the degree of DNA damage; intrinsic variability in the processes of DNA repair; or induction of SOS genes. Consequently, the potential growth‐dependence of the formation of DSBs and the subsequent induction of the DNA damage response may also have an impact on the heterogeneity of the SOS response, and subsequently on the development of antibiotic resistance. Population heterogeneity necessitates that we frame these questions at the single‐cell level.

In this study, we address how variation in growth‐rate modulated by nutrient quality influences the SOS expression in single cells under conditions of chronic sub‐lethal levels of DNA damage. In all conditions, we found a high degree of heterogeneity in SOS levels. We observed that cells with elevated SOS expression were more frequent in slow‐growth conditions. Using a microfluidic mother machine device we established that the rate of SOS induction is higher in fast‐growth conditions. This apparent contradiction can be explained by the competition between two distinct subpopulations in growing cultures: one with elevated SOS expression and very long division times; and another with moderate SOS expression and normal division times. Because division rates are highly dependent on nutrient conditions, the disparity in division times is much larger in fast‐growth condition, thus explaining the lower fraction of high SOS cells in the rich condition. Our observations highlight that the heterogeneity in division times is an important source of single‐cell variability in the DNA damage response and is likely to play a role in natural environments, where nutrient availability is highly variable.

## Results

### The fraction of cells with spontaneous high levels of SOS induction increases in slow‐growth conditions

As a baseline measurement of the DNA damage response in the absence of any artificial source of DNA damage, we characterized the steady‐state levels of SOS expression in cells grown in media with different nutrient composition. We quantified SOS induction using a transcriptional reporter based upon the well‐characterized SOS promoter *PsulA* driving the expression of *mGFP* and used fluorescence microscopy to measure the fluorescence‐per‐area as a proxy for GFP concentration (here referred to as “GFP intensity” expressed in arbitrary units of fluorescence per area; a. u./area) in more than 20,000 cells per condition (Dataset EV1). This transcriptional fusion was inserted in an ectopic chromosomal locus of a Wild Type *E. coli* strain (WT, MG1655) also carrying an *mKate* marker under the control of a constitutive promoter (*PtetO1*). To ensure that the population is in exponential growth (Schaechter, 2006), cells were grown for at least 12 generations with multiple dilutions before measurements were taken. We chose three growth conditions with population doubling‐rates for the WT strain as follows: 0.6 ± 0.01 (SEM) doublings per hour (M9‐glycerol, referred to as M9‐gly, slow‐growth conditions), 1.04 ± 0.04 doublings per hour (M9‐glucose, referred to as M9‐glu, medium‐growth conditions), and 1.61 ± 0.05 doublings per hour (M9‐glucose and amino acids, referred to as M9‐glu+aa, fast‐growth conditions). Importantly, in the fastest growth condition (with doubling every 37 min), cells undergo multi‐fork replication.

**Figure 1:**
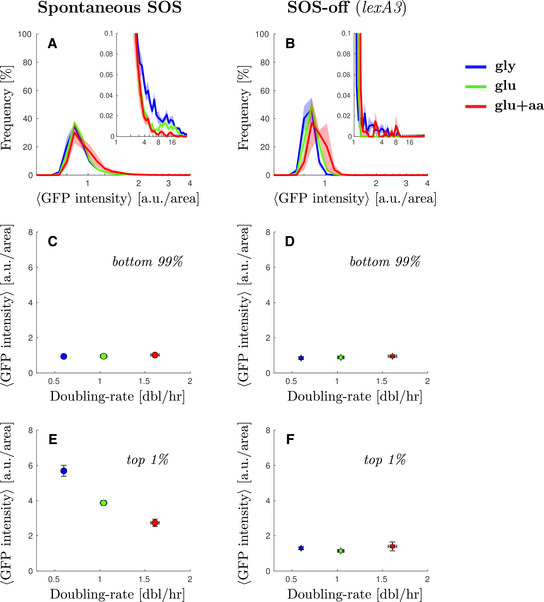
Spontaneous cells with elevated SOS expression are more frequent in low nutrient conditions Steady‐state distribution of GFP intensity from SOS reporter *PsulA‐mGFP* for WT cells in different growth conditions (blue M9‐gly, green M9‐glu, red M9‐glu+aa). GFP fluorescence intensity was measured as arbitrary units of fluorescence per unit cell area. Solid line represents the average frequency and shaded area the standard error from at least 3 replicates done on different days. Inset: A magnification of the second peak at high SOS expression.Steady‐state distribution of GFP intensity from SOS reporter *PsulA‐mGFP* for a strain unable to induce SOS (SOS‐off, *lexA3* background) in different growth conditions. Solid line represents the average frequency and shaded area the standard error from at least 3 replicates done on different days. Inset: A magnification of the high‐fluorescence range of the distribution.Average GFP intensity for the lower 99% of the population of WT cells as a function of growth rate. Points represent the average and bars the standard error from at least 6 biological repeats.Average GFP intensity for the lower 99% of the population of *lexA3* cells unable to induce SOS (SOS‐off, *lexA3* background) as a function of growth rate. Stars represent the average and bars the standard error from at least 3 biological replicates done on different days.Average GFP intensity for the top 1% of the population of wild type cells as a function of growth rate. Points represent the average and bars the standard error from at least three biological replicates done on different days.Average GFP intensity for the top 1% of the population of *lexA3* cells unable to induce SOS (SOS‐off, *lexA3* background) as a function of growth rate. Stars represent the average and bars the standard error from at least three biological replicates done on different days.

**Figure EV1:**
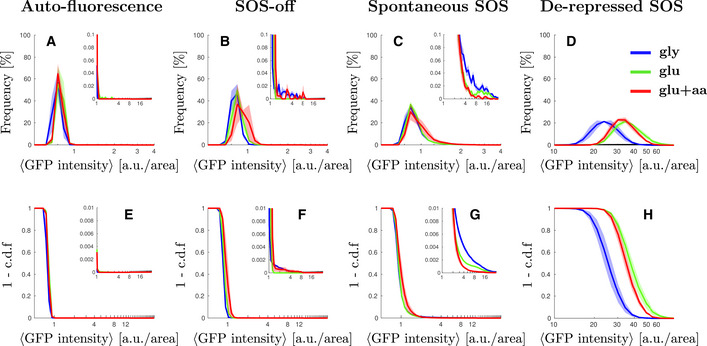
Single cell distributions of GFP intensity without inducing DNA damage For all plots, growth conditions are: M9‐glycerol (blue), M9‐glucose (green), and M9‐glucose+amino‐acids (red). Solid lines represent the average and shaded area the standard error from three biological repeats. Inset: A magnification of the second peak at high SOS expression.
Steady‐state distribution of GFP intensity from cell auto‐fluorescence in different growth conditions.Steady‐state distribution of GFP intensity from SOS reporter *PsulA‐mGFP* for cells unable to induce SOS (SOS‐off, *lexA3* background) in different growth conditions. Figure panel reused from Fig 1B for comparison purposes.Steady‐state distribution of GFP intensity from SOS reporter *PsulA‐mGFP* for wild type cells in different growth conditions. Figure panel reused from Fig 1A for comparison purposes.Δ*lexA* Δ*sulA* background.Steady‐state cumulative distribution of GFP intensity from cell auto‐fluorescence in different growth conditions.Steady‐state cumulative distribution of GFP intensity from SOS reporter *PsulA‐mGFP* for cells unable to induce SOS (SOS‐off, *lexA3* background) in different growth conditions.Steady‐state distribution of GFP intensity from SOS reporter *PsulA‐mGFP* for wild type cells in different growth conditions.Δ*lexA* Δ*sulA* background.

As expected, in the absence of external DNA damage, most cells do not show any SOS induction. The main peak of GFP intensity (as measured by *PsulA‐mGFP*; Fig 1A) is almost indistinguishable from the GFP intensity in a strain that is unable to induce SOS (Fig 1B. In this “SOS‐off” strain, the *lexA3* mutation makes LexA uncleavable (Lin & Little, 1988)). In addition, SOS expression for most of the population is close to the level of GFP auto‐fluorescence in our imaging conditions (approximately 15–20% difference), consistent with strong repression by LexA acting on the *PsulA* promoter (Fig EV1).

**Figure EV2:**
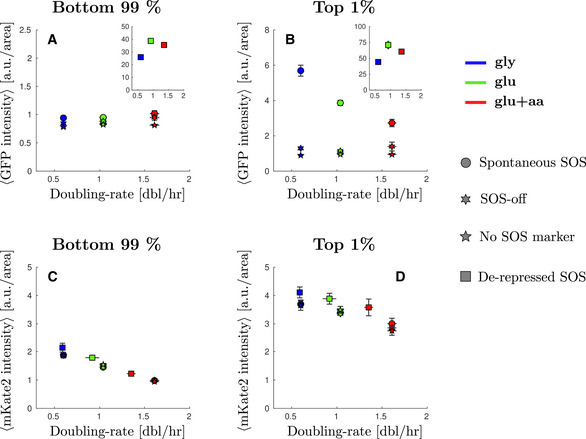
Average GFP and mKate2 intensity without inducing DNA damage For all plots, growth conditions are: M9‐glycerol (blue), M9‐glucose (green), and M9‐glucose+amino‐acids (red). Points (dots WT strain, 6‐points‐stars SOS‐off, *lexA3* background, 4‐point‐stars, autofluorescence) represent the average and bars the standard error from at least three biological repeats.
Average GFP intensity from SOS reporter *PsulA‐mGFP* for the bottom 99% of the population in different growth conditions. Inset: average GFP intensity in different growth conditions where SOS is de‐repressed.Average GFP intensity from SOS reporter *PsulA‐mGFP* for the top 1% of the population in different growth conditions. Inset: average GFP intensity in different growth conditions where SOS is de‐repressed.Average mKate2 intensity from constitutive reporter *PtetO‐mKate2* for the bottom 99% of the population in different growth conditions.Average mKate2 intensity from constitutive reporter *PtetO‐mKate2* for the top 1% of the population in different growth conditions.

We noticed, however, that the WT strain has a small subpopulation of highly expressing cells (Fig 1A, inset) that is not present in the “SOS‐off” mutant (Fig 1B, inset). Both the magnitude and the location of this secondary peak exhibit strong growth rate dependence. Comparing the blue curve (M9‐gly, slow‐growth conditions) with the red curve (M9‐glu+aa, fast‐growth conditions) in Fig 1A (inset), the fraction of cells in the high SOS state is approximately 6‐times larger in slow‐growth conditions compared with fast growth conditions (cells above 5 a.u./area of GFP intensity represent 0.3 and 0.05% for the blue and red curves, respectively). We compared the average SOS expression for the highest‐expressing 1% of the WT population across growth conditions and observed that the expression level is higher in the slow‐growth condition (5.9 ± 0.4 a.u./area) compared with fast‐growth condition (2.7 ± 0.2 a.u./area) (Figs 1E and EV2B). In contrast, there is no growth dependence in the expression level for the bottom 99% of the population (Figs 1C and EV2A). In the “SOS‐off” strain, we found no significant high‐expression peak across growth conditions; thus, the presence of high SOS cells in the wild‐type data is not due to leakiness from the *PsulA* promoter (Fig 1D and F). In other words, under conditions of spontaneous damage, SOS‐induced cells are more abundant in slow‐growth conditions, contrary to the expectation that in fast‐growth conditions cells may experience more damage due to their higher frequency of DNA replication (because of multi‐fork replication).

### The fraction of cells showing high levels of SOS expression induced by replication‐dependent DSBs increases in slow‐growth conditions

Spontaneous DNA‐damaging events are rare; to further evaluate the influence of growth‐conditions on SOS expression, we induced chronic artificial DNA damage. We chose to focus on SOS induction under constant sub‐lethal levels of DNA‐damage, which is commonly occurring in natural conditions (Kuzminov, 1999; Andersson & Hughes, 2014). We used a genetic system that mimics natural replication‐dependent breaks based upon the site‐specific cleavage of palindromic sequences inserted in the bacterial chromosome (Eykelenboom *et al*, 2008; Cockram *et al*, 2015; Amarh *et al*, 2018). Replication‐dependent DSBs at a single locus have been shown to have a minimal effect on the growth rate in rich nutrient conditions (reported doubling time 19.7 ± 1.2 min versus 19.6 ± 0.8 min for DSB versus WT strain, Darmon *et al*, 2014), and lead to low levels of SOS induction (Darmon *et al*, 2014). To generate moderate levels of SOS induction, we inserted two palindromes (located at opposite arms of the chromosomes) on the chromosome of an *E. coli* K12 strain containing the *PsulA‐mGFP* SOS transcriptional reporter (Materials and Methods).

**Figure 2:**
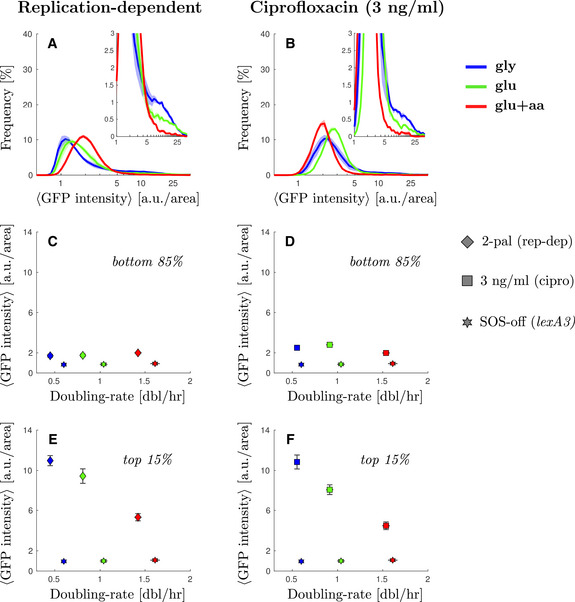
Cells with elevated SOS expression induced by DNA damage are more frequent in low nutrient conditions Steady‐state distribution of GFP intensity from SOS reporter *PsulA‐mGFP* when inducing replication‐dependent DSBs (2‐pal) in different growth conditions. Solid line represents the average frequency and shaded areas the standard error from at least three replicates done on different days. See Appendix Fig S3A and B for the comparable single‐palindrome data. Inset: A magnification of the second peak at high SOS expression.Steady‐state distribution of GFP intensity from SOS reporter *PsulA‐mGFP* under ciprofloxacin treatment (3 ng/ml) in different growth conditions. Solid line represents the average frequency and shaded area the standard error from at least 3 replicates done on different days. Inset: A magnification of the second peak at high SOS expression.Average GFP intensity for the lower 85% of the population under replication‐dependent DSBs (2‐pal) as a function of growth rate. Diamonds represent the average and bars the standard error from at least three biological replicates done in different days. For comparison, the SOS‐off (*lexA3*) mutant data is shown as stars.Average GFP intensity for the lower 85% under ciprofloxacin treatment (3 ng/ml) as a function of growth rate. Squares represent the average and bars the standard error from at least three biological replicates done in different days. For comparison, the SOS‐off (*lexA3*) mutant data is shown as stars.Average GFP intensity for the top 1% of the population under replication‐dependent DSBs (2‐pal) as a function of growth rate. Points represent the average and bars the standard error from at least three biological replicates done on different days.Average GFP intensity for the top 1% under ciprofloxacin treatment (3 ng/ml) as a function of growth rate. Points represent the average and bars the standard error from at least three biological replicates done on different days.

As with the spontaneous damage in the WT, we observed that SOS levels induced by replication‐dependent DSBs were highly heterogeneous in single cells. The SOS levels for the majority of the population showed only a moderate induction, as expected from the occurrence of at most two DSBs occurring only once per cell cycle (Fig 2A). Comparing slow‐growth with fast‐growth conditions, we observed that the main peak of the distributions shifts slightly to the right in faster‐growth conditions indicating higher levels of SOS expression for the bulk of the population (Fig 2A and Appendix Fig S1C). The average value for the main population (bottom 85% of the cells) increases slightly (diamonds, Fig 2C, 1.7 ± 0.2 a.u./area in M9‐gly against 2.01 ± 0.08 a.u./area in M9‐glu+aa). This moderate shift towards higher SOS levels for the main population is consistent with the higher number of replication forks in fast‐growth conditions leading to more replication‐dependent DSBs. This moderate increase may seem low compared with the sharp increase in the number of replication forks in rich nutrient condition but it should be noted that these DSBs occur in a context where they are also more likely to be repaired rapidly (thus potentially limiting SOS induction) as more homologous DNA copies are available. We also observed higher SOS values in the strain containing both palindromes than in the strains with a single palindrome consistent with a higher number of DSBs in the double palindrome strain (Appendix Fig S1A and B).

**Figure EV3:**
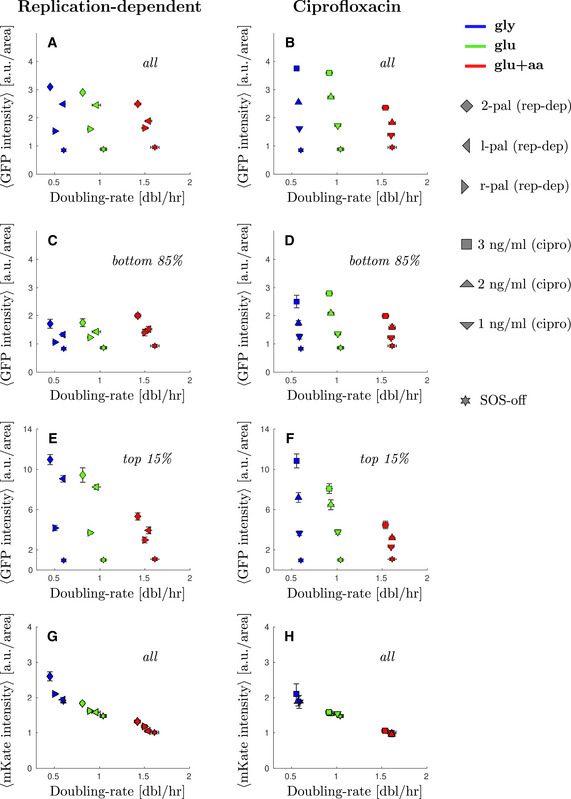
Average GFP and mKate2 intensity from inducing double‐strand breaks For all plots, growth conditions are: M9‐glycerol (blue), M9‐glucose (green), and M9‐glucose+amino‐acids (red). Points represent the average and bars the standard error from at least three replicates done in different days.
Average GFP intensity from SOS reporter *PsulA‐mGFP* for the whole population under replication‐dependent DSBs as a function of growth rate.Average GFP intensity from SOS reporter *PsulA‐mGFP* for the whole population under ciprofloxacin as a function of growth rate.Average GFP intensity from SOS reporter *PsulA‐mGFP* for the bottom 85% of the population under replication‐dependent DSBs as a function of growth rate.Average GFP intensity from SOS reporter *PsulA‐mGFP* for the bottom 85% of the population under ciprofloxacin as a function of growth rate.Average GFP intensity from SOS reporter *PsulA‐mGFP* for the top 1% of the population under replication‐dependent DSBs as a function of growth rate.Average GFP intensity from SOS reporter *PsulA‐mGFP* for the top 1% of the population under ciprofloxacin as a function of growth rate.Average mKate2 intensity from constitutive reporter *PtetO‐mKate2* for the whole population under replication‐dependent DSBs as a function of growth rate.Average mKate2 intensity from constitutive reporter *PtetO‐mKate2* for the whole population under ciprofloxacin as a function of growth rate.

As with spontaneous damage, we observed a “shoulder” of high SOS‐expressing cells, with a larger fraction of cells in the high SOS state in slow‐growth condition (Fig 2A, inset). Gaussian fitting of the distributions (Appendix Fig S8) are indicative of the potential presence of two populations with the average of the high‐SOS population (varying between 2.27 and 2.9 a.u./area depending on growth conditions) being twice that of low‐SOS population. We chose to set a high cut‐off to define the second population so that the high‐SOS population is well‐discriminated in all growth conditions. In the rest of the analysis, we focus on the fraction of cells whose fluorescence intensity is above 5 a.u./area. This fraction is 14 ± 1% in M9‐gly versus 4.3 ± 0.7% in M9‐glu+aa. To visualize more clearly this phenomenon, we measured the average SOS levels in the high SOS fractions of the population (Fig 2C and E). The average SOS level for the top 15% cells of each population shows a clear negative correlation with population growth rate (Fig 2E, 11.0 ± 0.5 a.u./area in M9‐gly against 5.3 ± 0.2 a.u./area in M9‐glu+aa). The SOS levels for the top fraction of the population were also found to be higher in slow‐growth conditions for strains carrying single palindromes (Fig EV3E, Appendix Fig S1). This result is unexpected given the positive correlation of SOS levels with growth rates for the main population, suggesting that the high SOS induced population might behave differently than the rest of the population. A potential explanation could be that in the fraction of the population where SOS is derepressed, an SOS regulated protein behaves similarly to a constitutive stable protein and its concentration is therefore negatively correlated with growth rate (Scott *et al*, 2010), as we observe for a constitutively expressed mKate protein (Figs EV2C and D, and EV3G). If this were the main explanation, we would expect a negative correlation of GFP driven by an SOS promoter with growth when SOS is fully derepressed as in a Δ*lexA* mutant. This is not the case: we observe a *lower* concentration at low growth rate compared with intermediate and fast growth rates (Fig EV1D and H and insets Fig EV2A and B). Therefore, our data suggest that the simple growth dependence predicted by the “bacterial growth laws” is not sufficient to explain our observations.

### The fraction of cells showing high levels of SOS expression induced by exposure to ciprofloxacin increases in slow‐growth conditions

To test the generality of our observations, we performed a similar experiment by inducing DSBs using sub‐lethal concentrations of a fluoroquinolone, ciprofloxacin (Chen *et al*, 1996; Tamayo *et al*, 2009). As expected, the majority of the population showed a moderate induction in SOS expression, which increased with the concentration of ciprofloxacin (1–3 ng/ml; Fig 2B and Appendix Fig S2). The level of induction for the main population did not show any clear growth dependence suggesting that changes in the number of replication forks may not directly relate to the frequency of DNA damage under such low levels of ciprofloxacin exposure. It is also possible that the uptake (or efflux) of the drug is specifically affected in one of the growth media which is why subsequent mother machine experiments were carried out with the replication‐dependent DSB system.

Nonetheless, as with replication‐dependent DSBs, we observed growth‐dependent heterogeneity in the response. Indeed, a fraction of the population reached very high SOS induction and this fraction was larger in poor than in rich nutrient conditions (Fig 2B, inset, and Appendix Fig S8). For example, 13 ± 2% of the cells showed a fluorescence intensity above 5 a.u./area in M9‐gly versus 2.4 ± 0.3% in M9‐glu+aa. Furthermore, under exposure to 3 ng/ml of ciprofloxacin, we observed that the average SOS level for the top 15% cells of each population shows again a clear negative correlation with population growth rate (10.9 ± 0.7 a.u./area against 4.5 ± 0.4 a.u./area comparing the red and blue squares). In contrast, the bottom 85% shows negligible growth rate correlation (2.5 ± 0.2 a.u./area against 2.00 ± 0.06 a.u./area comparing the red and blue squares). Similar trends were observed for intermediate doses of ciprofloxacin (Appendix Fig S2 and Fig EV3). Therefore, we conclude that, consistent with the other mechanisms of DNA damage studied, exposure to ciprofloxacin leads to a subpopulation of cells with high SOS induction that behave differently from the rest of the population with respect to growth‐rate change.

### Cells with high levels of SOS induction arrest division

The data presented so far indicate that, independently of how DSBs are induced, the fraction of cells with high SOS expression is higher in slow‐growth conditions. To better understand the dynamic interplay between growth conditions and SOS induction by DNA damage, we used time‐lapse microscopy and observed single‐cells growing on agar pads for about eight divisions. As expected, we observed only a small fraction of cells inducing very high levels of SOS expression (Appendix Figs S3 and S4). The majority of cells with very high SOS levels delayed or stopped division, which is consistent with the induction of the SOS‐dependent cell division inhibitor SulA (Huisman *et al*, 1984; Cambridge *et al*, 2014; Burby & Simmons, 2019). In contrast, cells with more moderate levels of SOS induction went through several rounds of division during the course of the experiment.

Thus, our time‐lapse data suggest that there are two subpopulations present with respect to cell division: one that divides normally with relatively low or intermediate SOS expression levels, and a second that divides very slowly with high SOS expression levels. This has direct consequences on the relative abundance of each type of cell in a growing population because high SOS cells might be partially out‐competed by the rest of the (more rapidly‐dividing) population.

### The transition rate to high‐SOS state is higher in fast‐growth conditions

**Figure 3:**
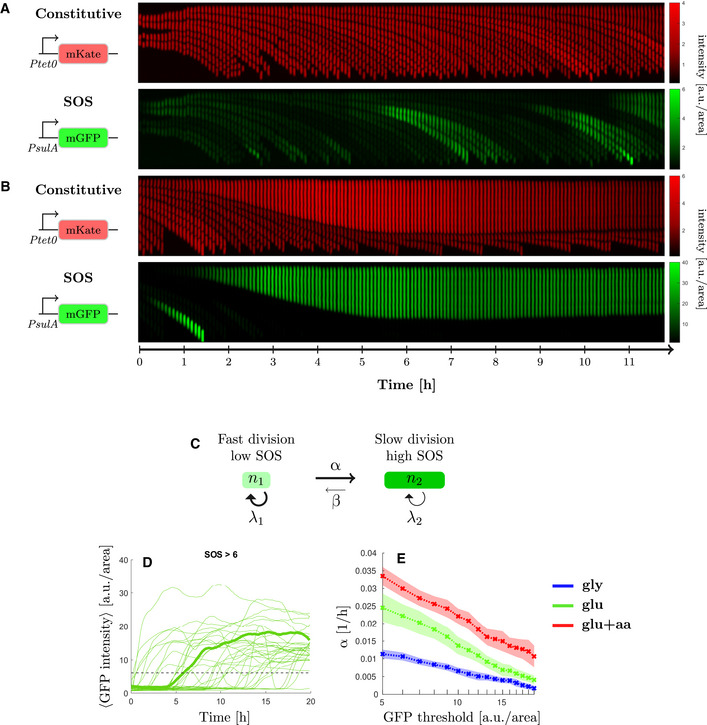
The transition rate to high‐SOS state is higher in fast‐growth conditions Representative kymographs of a strain experiencing replication‐dependent DSBs (2‐pal) in M9‐glucose+amino‐acids medium. Scale bars represent normalised intensity for constitutive reporter *PtetO‐mKate2* and GFP intensity from SOS reporter *PsulA‐mGFP*.
Example mother cell lineage with low level of SOS induction. Top, constitutive expression of mKate, bottom *PsulA‐mGFP*.Example mother cell lineage inducing a high level of SOS. Top, constitutive expression of mKate, bottom *PsulA‐mGFP*. Cell division is inhibited while cell growth continues and eventually stops.Switching model. Cells with low levels of SOS induction rate switch at rate α to a high SOS level state and switch back at rate β (with β≪α).GFP intensity trajectories of SOS induction observed in the 2 palindrome strain as a function of time. One trajectory has been highlighted in bold. The dashed line corresponds to the threshold discriminating between high and low‐SOS cells.Switching rate estimated for multiple GFP intensity thresholds under replication‐dependent DSBs (2‐pal) in different growth conditions (see Appendix Supplementary Methods for the estimation). The switching rate is always higher in rich than in poor nutrient. Points represent the average and the shaded area the standard error from three biological repeats.

**Figure EV4:**
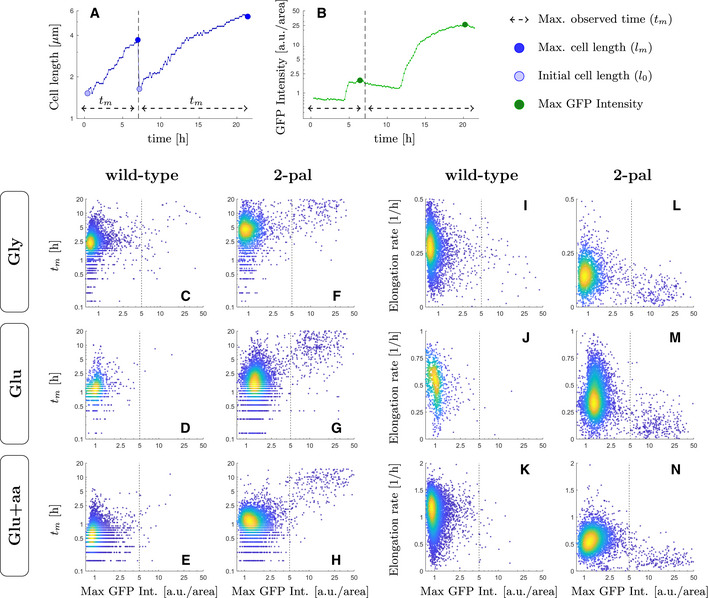
High‐levels of SOS expression correlates with reduction in cell division and elongation rate under replication‐dependent DNA‐damage For every single‐cell trajectory four metrics were computed as proxies for division and elongation rate reduction and its relation to GFP levels: the maximum observed time before a division or end of the tracking (*t_m_*), the minimum and maximum cell lengths (*l_0_* and *l_m_*), and the maximum GFP intensity registered for that lineage. A, BThese metrics are illustrated for a lineage with two single‐cell trajectories.C–HThe maximum observed time (*t_m_*) was plotted against the maximum GFP intensity, comparing wild‐type and cells undergoing replication‐dependent DNA‐damage (2‐pal), where extreme values of GFP intensity correlate with significant delays in cell division only for the 2‐pal strain.I–NWe plot a proxy for the cell elongation rate computed as log(*l_m_*− *l_0_*)/*t_m_* against the maximum GFP intensity, where extreme values in GFP intensity correlate with a reduction in elongation rate as compared to the rest of the population. Data information: Throughout panels C to N, colours represent the 2D density for each individual dataset, where yellow and blue denote high and low density, respectively. The vertical dashed line denotes the cutoff used throughout to discriminate high levels of SOS induction. Panels C‐H contains data from 4,340, 1,016, 7,393, 2,572, 5,978, and 9,880 cell cycles, respectively.

To better understand the dynamics of the induction of low and high SOS levels in cells independently of the competition that arises from the differences in division rates of these two states, we made use of a microfluidic mother machine (Wang *et al*, 2010). In this set‐up, each cell is trapped in its individual channel enabling measurements of real‐time SOS induction and division rates. We collected fluorescence images of the strain carrying the 2 palindromes over 10–40 h in the three growth media used previously. We used the constitutively expressed *mKate2* marker for cell segmentation and detection of division and the *PsulA‐mGFP* marker to monitor SOS induction. Consistent with our observation on agar pads, we observed cell lineages that induced moderate levels of SOS and continued to divide (Fig 3A) as well as lineages where SOS induction was higher and cell division was strongly delayed or arrested, leading to the formation of filaments (Fig 3B). These experiments were performed at least three times in each growth condition. The distributions of division time, cell elongation rate and fluorescence intensity are shown in Appendix Fig S5 and show good day‐to‐day reproducibility. Next, we examined the correlation between the maximum SOS induction, the maximum time the cell was observed (i.e. birth and division or birth and the time the experiment was completed if the cell did not divide) and the cell elongation rate. As shown in Fig EV4, in the strain that carries the 2 palindromes, we observed two populations of cells. The main population showed relatively low levels of SOS induction (with GFP/area below 3 a.u./area) and normal elongation rates (ranging from 0.5 h^−1^ to 0.12 h^−1^ depending on the growth conditions) A second population reached high SOS level of at least 5 a.u./area in all growth conditions (Fig EV4F, G, H, L, M and N), which correlated with a much higher observation time (at least 5 to up to 20 h, corresponding to very low division rates or no division in the course of the experiment) and lower elongation rate of < 0.1 h^−1^. The fraction this second population corresponds to 7.89% in M9‐gly, 4.58% in M9‐glu and 3.4% in M9‐glu+aa. This confirms the existence of two populations with markedly different behaviours with respect to SOS induction and division rates. In contrast, the WT strain had very few (between 0.43% in M9‐gly and 0.014% in M9‐glu‐aa) outlier cells confirming that our observation is due to the presence of DNA damage.

To account for the dynamic equilibrium of the high and low SOS populations during exponential growth, we adapted a previous mathematical model used to explain the population dynamics of persister cells (Balaban *et al*, 2004; Patra & Klumpp, 2013). In this model, cells can be in two states (low and high SOS, Fig 3C), corresponding to different division rates (λ_1_ and λ_2_, with λ_1_>λ_2_). The total number of cells in the population is fixed, as we follow only mother cells in the microfluidics device, and cells can switch from low to high SOS at rate α (switching back at rate β). An example of time traces of cell trajectories for expression of the *PsulA‐mGFP* is shown in Fig 3D: we recorded the time at which each cell reached a threshold of GFP intensity above 5 a.u./area to estimate the rate at which cells induce high level of SOS. Very few cells reverted from high to low SOS, and when they divided they produced a single small cell as previously reported (Raghunathan *et al*, 2020), so we consider β to be negligible and do not consider it in our model. We used Maximum Likelihood estimation (see Appendix Supplementary Methods) to compute α for GFP thresholds ranging from 5 to 20 a.u./area in the three growth media (Fig 3E and Appendix Fig S6). As seen in Fig 3E, the rate of switching to high SOS is always higher in rich nutrient condition than in poorer ones irrespective of the threshold, although 5 a.u./area is the threshold that gives the highest discriminatory power. This indicates either that individual cells have a higher probability per unit of time of switching to high SOS in rich nutrient conditions (in keeping with the higher number of replication forks in these conditions) or that the response time of SOS induction is simply driven by the generation time as described in Alon (2007) and is therefore faster in rich nutrient conditions. In conclusion, the larger fraction of high SOS cells that we observed in slow, exponentially growing populations is *not* explained by a higher rate of SOS induction. Rather, it is the result of a competition between high SOS/slow‐dividing and low SOS/fast‐dividing cells.

### A mathematical model of an expanding population explains the large fraction of high SOS cells observed in slow growth conditions in batch cultures

**Figure 4:**
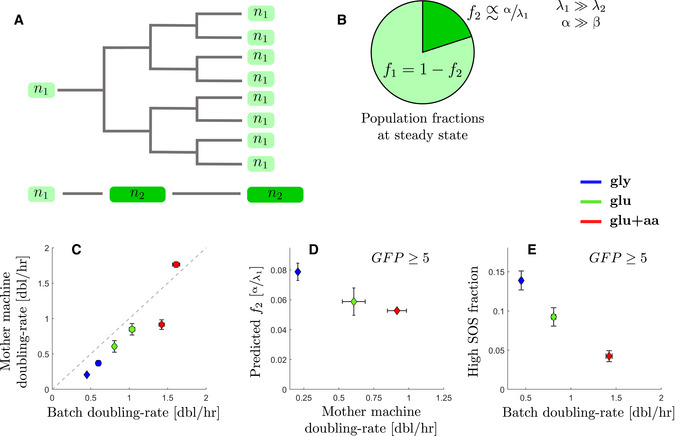
A two‐populations model explains the large fraction of high SOS cells observed in slow growth conditions Population model: Cells with high levels of SOS induction slow cell division and are outcompeted, in terms of cell numbers, by lineages experiencing low levels of SOS induction.In conditions when rate of switching to high SOS is much higher that the reverse and where the high SOS cells divide very slowly, the population fraction of cells with high SOS levels is expected to be inversely proportional to the growth‐rate λ_1_ of the subpopulation with low SOS levels (at steady‐state).Comparison between doubling rates for the WT (circles) and 2‐pal mutant (diamonds) estimated from growth in the mother machine and in batch in different growth conditions. Mother machine doubling rates are the estimated population doubling rate derived from the underlying single‐cell division‐rate distribution. Points represent the average and bars the standard error from at least 3 biological repeats.Predicted steady‐state population fractions for high SOS cells (with a threshold of 5 arbitrary units) from the SOS reporter *PsulA‐mGFP* under replication‐dependent DSBs (2‐pal) in different growth conditions. Points represent the average and bars the standard error from three biological repeats. The data are the prediction using rates estimated from mother machine experiments.Batch population fractions of high SOS cells (above 5 arbitrary units) from the SOS reporter *PsulA‐mGFP* under replication‐dependent DSBs (2‐pal) in different growth conditions. Points represent the average and bars the standard error from three biological repeats.

To better understand the interplay between the rate of SOS induction and the division rates of low and high SOS cells, we expanded our model above to describe a growing population (Fig 4A). Given that the rate of switching from high SOS to low SOS (β) is negligible and that the division rate of high SOS cell (λ_2_) is small, we can show that the expected fraction of high SOS cell (*f*_2_) is approximated by $f_{2}\approx \frac{\alpha }{\lambda_{1}}$ (Fig 4B and Appendix equation (7)). We estimated the division rates of the low SOS cells (λ_1_) from the mother machine experiments using previously established methods (Painter & Marr, 1968; Thomas, 2017) (Appendix Fig S12 and Appendix Supplementary Methods). We observed that the population growth rate in the mother machine was similar to the batch experiment in M9‐glu‐aa for the WT strain but lower in M9‐gly and M9‐glu by approximately 20% (Fig 4C) possibly due to slight constriction of the cells in the mother machine device (Yang *et al*, 2018). This trend was more pronounced in the strain carrying two palindromes (with a decrease of almost 50% in M9‐glu‐aa) which may be due to over‐estimation of the batch growth rate due to the impact of filamentation on OD measurements (Stevenson *et al*, 2016). To test the validity of this explanation, we performed growth rate measurements in batch cultures using Colony‐Forming Units (CFU) instead of OD. We observed that the growth rates estimated from CFUs were similar to those obtained with the mother machine experiments (Appendix Table S1, Appendix Fig S7). In particular, for the strain carrying 2 palindromes in M9‐glu‐aa, we obtained a doubling rate of 1.08 dbl/h in CFU based batch measurement, compared with 0.98 dbl/h in mother machine experiment (but lower than the 1.42 dbl/h obtained by OD measurements). Therefore, we conclude that OD measurements lead to over‐estimation of growth rate especially when cells experience DNA damage.

When computing the expected fraction of high SOS cells (*f*_2_), we observed that our model predicts a higher fraction of high SOS cells in poor nutrient conditions, despite lower rate of SOS induction (Fig 4D). For example, the expected fraction of cells reaching a GFP fluorescent intensity of at least 5 a.u./area is 7.81 ± 0.6% in M9‐gly, 5.92 ± 0.9% in M9‐glu and 5.25 ± 0.1% in M9‐glu+aa. This is similar to the trend we measured in batch experiments (Fig 4E, respectively, 14 ± 1% in M9‐gly, 9 ± 1% in M9‐glu and 4.3 ± 0.7% in M9‐glu+aa). The prediction is in very good agreement with the observed proportion of high SOS cells in rich nutrient conditions, especially when taking into account the over‐estimation of the growth rate in batch OD based experiments (we kept OD based growth rate in Fig 4E as CFU were not measured in these experiments). However, our model underestimates the fraction of high SOS cells in low nutrient conditions. For example, we observe 14 ± 1% cells above 5 a.u./area versus a prediction of 7.81 ± 0.6% in M9‐gly. To explain this discrepancy, we used a more precise estimate of the fraction of high SOS cells (*f*_2_) in low nutrient conditions where the division rate of high SOS cells (λ_2_) likely cannot be neglected as the low SOS cells are also dividing slowly. Therefore, a better estimate for *f*_2_ would be $f_{2}\approx \frac{\alpha }{\lambda_{1}}\left(\frac{1}{1‐\frac{\lambda_{2}}{\lambda_{1}}}\right)$ (Patra & Klumpp, 2013; Appendix equation 8). In the mother machine experiments, we observed a limited number of high‐SOS cells’ divisions across all growth conditions, and thus estimated for $\lambda_{2}\approx 0.1/h$ from Fig EV4. Using this approximation and the equation above, we can correct the prediction for *f*_2_ which gives ≈15% for M9‐gly and ≈7.1% for M9‐glu. These values are closer to the fraction observed in the batch experiments indicating that in low nutrient conditions we cannot fully neglect the contribution of the division of the high SOS cells. Thus our model explains the counter‐intuitive result that high SOS cells are more frequent in poor nutrient conditions despite the higher rate of SOS induction in fast‐growth conditions.

## Discussion

In natural environments, bacteria are exposed to varying levels of nutrient availability and subject to sub‐lethal stresses (Andersson & Hughes, 2014). The induction of stress responses, such the DNA damage response (SOS), consequently plays an important role in survival. In this paper, we show that heterogeneity in the levels of SOS expression induced by chronic sub‐lethal DNA‐damage exhibits strong growth‐dependence. Surprisingly, we observe a larger fraction of highly induced cells in poor nutrient conditions despite a higher rate of SOS induction in rich nutrient conditions. This counter‐intuitive result can be explained by the dynamic balance between the rate of SOS induction and the attenuated division rates for high‐SOS induced cells.

At the single‐cell level, SOS induction decouples growth from division. Our results indicate that this decoupling has a major impact on the dynamics of the population. Classically, during balanced growth, the population doubling rate (µ) is directly related to the exponential rate of mass accumulation (λ = ln2*µ) (Monod, 1949). When cells induce the SOS response, they become filamentous because they continue adding mass whilst delaying division as a result of the induction of SulA, YmfM and potentially other division inhibitors (Ansari *et al*, 2021). Heterogeneity in the level of SOS induction produces strong heterogeneity in the division rates of individual cells in the population. As we have shown, this leads to counter‐intuitive effects at the population level, where the frequency of high SOS cells is not simply the result of the rate of SOS induction but also depends upon competition with the rapidly‐proliferating non‐induced cells.

Our work highlights the importance of a multi‐scale approach to the analysis of bacterial stress responses. In the case of sub‐lethal DNA damage, analysis of the population‐averaged SOS induction level shows a negative growth‐dependence, with higher induction in poor‐nutrient conditions than in rich‐nutrient ones (Fig EV3A and B). A negative correlation between expression level and growth‐rate is consistent with the growth‐dependence of a fully induced protein, and could suggest that rich nutrient conditions impose limits on SOS induction to express the requisite translation machinery (Scott *et al*, 2010; Hui *et al*, 2015; Weiße *et al*, 2015). Analysis at the single cell level, however, shows a somewhat different picture. For the majority of the population (bottom 85%, Fig 2C and D), we do not observe any growth dependence of the expression level of SOS genes. This could be explained by the negative auto‐regulation of LexA at low level of SOS induction; negative feedback results in homeostatic expression levels, abrogating any intrinsic growth‐rate dependence (Klumpp *et al*, 2009). For the high SOS cells (top 15%, Fig 2E and F), we do observe an average negative growth‐dependence in the expression level of SOS genes, consistent with physiological constraints characterized in balanced growth. The dynamic equilibrium of these phenotypically distinct subpopulations is maintained by a balance between the rate of SOS induction (α in our model) and the growth rates of the two subpopulations (Fig 3C). Although the growth‐dependence of the expression of SOS genes in each subpopulation conforms to what is known from physiological constraints on gene expression, our understanding of the population behaviour critically depends upon quantification of the dynamics of molecular processes at the single‐cell level via the SOS induction rate α.

The induction of the SOS response is known to have multiple consequences beyond facilitating the repair of DNA (Podlesek & Žgur Bertok, 2020), including increasing antibiotic tolerance (Dörr *et al*, 2009; Wu *et al*, 2015), modulating expression of mobile genetic elements (Beaber *et al*, 2004; Baharoglu *et al*, 2010; Fornelos *et al*, 2016), and increasing the rate of mutagenesis (Vaisman *et al*, 2012; Dapa *et al*, 2017; Pribis *et al*, 2019). Our observed growth‐dependent heterogeneity in the fraction of SOS‐induced cells suggests that care must be taken when making quantitative estimates of the mutation rate under conditions of sub‐lethal DNA damage. For example, if the majority of mutants generated in a fluctuation assay arise from the high‐SOS fraction, then the inferred mutation rate must be corrected (via multiplication by the reciprocal fraction of high‐SOS cells, 1/*f*_2_) to account for the small subpopulation size. Quantitative predictions of the mutation rate in SOS‐induced cells could therefore be underestimated by a factor of 20 or more. Furthermore, the correction to subpopulation size is growth‐rate dependent (Fig 4E), introducing an inherent growth‐rate dependence in the calculation of the mutation rate. Given the growth‐dependent heterogeneity in the population under SOS‐induction, microfluidic single‐cell mutation‐accumulation assays (Robert *et al*, 2018; Uphoff, 2018) offer a useful tool to deconvolve heterogeneous stress response from downstream genetic change.

We have shown that single cell heterogeneity in division times can have important consequences in the abundance of cells with high SOS levels in growing populations. Similar heterogeneous population dynamics have been used to describe the maintenance of persister fractions (Balaban *et al*, 2004; Patra & Klumpp, 2013), and to describe phase‐transitions in the stability of antibiotic‐resistant strains (Deris *et al*, 2013; Roy & Klumpp, 2018). In addition to DNA damaging antibiotics, a growth‐dependent heterogeneous response is likely to occur in treatments with cell‐wall targeting antibiotics, as they can inhibit cell division, induce moderate levels of the SOS response, and induce the general stress response (Miller *et al*, 2003; Laureti *et al*, 2013; Lambert & Kussell, 2015). More generally, our results argue that whenever a stress leads to a transition towards a non‐dividing or slow‐dividing state (in our case high SOS expression), the fraction of these cells will be enriched in slow‐growth conditions. This is true even though the stress response is often faster in fast growing condition as the response time of a gene expression system often simply scales with the generation time (Alon, 2007). This phenomenon is likely to affect the fraction of subpopulations in natural environments with varying levels of nutrient availability, and introduces an intrinsic growth‐rate dependence in bet‐hedging strategies (Veening *et al*, 2008). Growth‐rate dependent heterogeneity under DNA‐damaging conditions introduces an additional degree of freedom in the complex coupling between the growth environment and evolutionary change.

## Materials and Methods

### Culture conditions

For all microscopy and batch experiments, cell cultures were grown in M9‐based media. The composition of the M9 salts was as follows: 49 mM Na_2_HPO_4_, 22 mM KH_2_PO_4_, 8.6 mM NaCl, 19 mM NH_4_Cl, 2 mM MgSO_4_, and 0.1 mM CaCl_2_ (adjusted to pH = 7). This was supplemented with either 0.5% w/v glycerol or 0.5% w/w glucose, and a mix of amino acids (1× MEM Non‐Essential Amino Acids and 1× MEM Amino Acids, both manufactured by Gibco™). For strains and plasmid construction, cells were grown in LB, or LB agar supplemented with the corresponding selection markers. Concentrations employed for antibiotics were as follows: ampicillin 100 μg/ml, kanamycin 50 μg/ml, and gentamycin 10 μg/ml. All cultures were grown in 50 ml falcon tubes agitated at 37°C (300 rpm) with no more than 5 ml of liquid volume, unless otherwise stated.

Cell cultures were grown for at least 12 division times in each media to reach steady‐state exponential growth before taking measurements. This was carried out as follows. Cells were taken from frozen stocks at −80°C, and grown for 10–16 h in LB media. They were then grown overnight (after a 1:1,000 dilution) in their respective M9‐based media, plus appropriate antibiotics in case of a selection marker. These overnight cultures were diluted 1:200 in fresh media (without antibiotic), and grown until OD600 0.1 (approximately three division times). From there, they were diluted again in fresh media (with dilution in the order of 10^−5^–10^−6^) so that experiments could be performed the next day. These last dilution factors were calculated to allow for at least 12 division times (without reaching an OD600 higher than 0.15) before any measurement. During the day, samples were diluted when necessary to prevent reaching OD600 higher than 0.15. Population (batch) growth rates were estimated by OD600 measurements over time in three technical repeats and three biological repeats per condition.

Batch growth rates from colony‐forming units (CFUs) were determined as follows:

An overnight culture was prepared as described above. Measurements were taken every 15–30 min depending on the growth condition. For each measurement, the optical density (OD600) was read and then serial dilutions (in steps of 1:10) were performed in a 96‐well plates up to a dilution of 10^−6^. Sterile, pre‐warmed PBS was used as diluent. A total of 100 µl of cells were transferred to pre‐warmed LB agar plates and the culture was spread using approximately 10–15 sterile glass beads. The plates were allowed to dry before removing the beads and inverting. Plates were incubated at 37°C overnight. Plate counts were performed in duplicate for each serial dilution where possible. Colonies were counted and CFU/ml calculated for plates containing 30–300 CFUs. CFU‐derived growth rates were estimated via linear regression to the log‐transformed CFU/ml growth curves during the exponential growth phase.

### Strain and plasmid construction

*Escherichia coli* MG1655 was used as WT strain in this study. The strains and plasmids are listed in Appendix Tables S2 and S3, respectively. Gene‐expression reporters (GFP for SOS expression and mKate for constitutive gene expression) were cloned into pOSIP plasmids inserted into the genome by clone‐integration (St‐Pierre *et al*, 2013). Plasmid construction was performed by Gibson assembly after PCR amplification of the fragments (see Appendix Table S4 for the detailed description and Appendix Table S5 for the list of primers used). All strains were checked with PCR amplification followed by Sanger sequencing. Insertion of interrupted palindromes performed via P1 transduction using strains kindly given by D. L. Leach (see Appendix Table S2).

### Fluorescence microscopy

All images were captured using a Nikon Ti‐E inverted microscope equipped with EMCCD Camera (iXion Ultra 897, Andor), a SpectraX Line engine (Lumencor) and a 100× Nikon TIRF objective (NA 1.49, oil immersion). Nikon Perfect‐Focus system was used for continuous maintenance of focus. The filter set for imaging *mGFP* consisted of ET480/40× (excitation), T510LPXR (dichroic), and ET535/50m (emission); whereas for *mKate2* the set ET572/35× (excitation), T590LPXR (dichroic), and ET632/60m (emission) was used. Filters used were purchased from Chroma. GFP fluorescence was measured using 80 ms exposure, whereas mKate2 fluorescence was imaged for 100 ms, both at minimal gain and maximum lamp intensity. Microscope was controlled from MATLAB via MicroManager (Edelstein *et al*, 2014).

### Agar‐pad microscopy and image analysis

For agar‐pad microscopy, steady‐state exponential cell cultures were prepared as described previously. For imaging, samples were mounted on agar‐pads: 5–10 μl from cultures around OD600 0.05 were placed in 1% agarose pads (Gene‐frame 65 μl) made with the corresponding growth media. For each repeat, about 250 stage positions were imaged, comprising a total of 4,000–30,000 cells after image analysis. All conditions were performed in at least three biological repeats. For time‐lapse microscopy experiments, 2 μl from liquid cultures at balanced growth were placed in agar‐pads as described above. About 15 different stage positions were imaged at intervals lasting one‐tenth of the population doubling time, for one hundred time intervals.

To automate the detection of cells from fluorescent images, we developed an algorithm for edge‐detection using custom low‐pass filters (the algorithm is detailed in the Appendix Supplementary Methods and Appendix Fig S10). Results from the automated cell segmentation were manually curated to remove any misidentified cell and false positives. Fluorescence signal from the constitutive reporter *PtetO‐mKate2* was used in all cases for cell segmentation. All *mGFP* and *mKate* fluorescence values were re‐scaled by the average fluorescence value of the WT strain data sets in each growth medium. After cell segmentation, fluorescence signal concentration was quantified by summing the total intensity for each cell divided by the number of pixels. For the population frequency plots, data were binned and plotted in log‐scale intervals (the mean ± standard error across biological repeats is reported). Inter‐day variability is shown in Appendix Fig S9.

### Mother machine experiments

#### Microfluidics design and fabrication

We used a mother machine design similar to (Wang *et al*, 2010), consisting of an array of closed‐end microchannels connected to a large flow channel. The device was designed using OpenSCAD and the photomask was manufactured by Compugraphics International Ltd. The master moulds were produced at the Scottish Microelectronics Centre, Edinburgh, using standard soft lithography techniques and SU‐8 photoresists on a 4” silicon wafer. This was done in two steps: the first layer for the microchannels and the second layer for the flow channel. The length of the microchannels was ~25 μm and the height of the flow channel was ~22 μm. The size of *E. coli* cells change depending on the medium used ‐ this scales with growth rate. To accommodate for this, our design consisted of microchannels with a range of widths (from 0.9 to 1.9 μm) and several master moulds were fabricated corresponding to different heights (0.9–1.36 μm). Appropriate dimensions were tested and selected for each growth condition. See Appendix Table S6 for specific dimensions used. The poly‐dimethylsiloxane (PDMS) chips were made using the Silicone Elastomer Kit 184 (Sylgard, Dow Corning) with a 1:10 ratio of curing agent to base. The protocol used to fabricate the microfluidics chips is summarized in the Appendix Supplementary Methods.

#### Culture preparation

A total of 10 ml of cultures were grown into steady‐state exponential phase using similar pre‐culture conditions described earlier. Cells were harvested at OD600 ≈ 0.2 and concentrated 100‐fold by centrifugation (4,000 rpm for 5 min). Tween‐20 (Thermo Scientific Pierce) was added to the culture (0.01% final concentration) before centrifugation to prevent clumping. Before sample loading, the chip was passivated with Tween‐20 (0.01%) for at least 1 h. The concentrated cell culture was then injected into the feeding channels using a 1 ml syringe with a 21‐guage blunt needle (OctoInkjet). Cells were allowed to diffuse into the microchannels for approximately 30 min at 37°C. To further assist loading, the cells were then spun into the microchannels by centrifugation at 3,220 *g* for 5 min using a custom‐built mount. The microfluidics device was then mounted on the microscope and connected to a peristaltic pump (Ismatec IPC ISM932D) on one end, and to fresh media + 0.01% Tween‐20 on the other end, to flow fresh media through the device. Cells were flushed from the main trench at 1.5–2 ml/h and then the flow rate lowered to 1 ml/h for the duration of the experiment. Experiments were run for 10–42 h depending on the nutrient conditions. Cells in the channels were allowed to recover for at least 2 h at 37°C before imaging.

#### Microscopy and image analysis

For mother machine experiments, images were acquired at 5 min intervals for M9‐glu+aa, 10 min for M9‐glu, and 12 min for M9‐gly. Images were saved in.mat format as one file per fluorescence channel per frame. The images were then converted to TIFF format in MATLAB. Segmentation and tracking were performed using BACMMAN run in Fiji (Ollion *et al*, 2019). The BACMMAN configuration was adapted to segment and track cells based on fluorescence images. Images were imported in BACMMAN and pre‐processed prior to segmentation. This included image rotation to ensure channels were vertically oriented and the channel opening was at the bottom, and cropping of images to include only the area consisting of microchannels. Joint segmentation and tracking of cells were performed on the *mKate2* fluorescence channel. Segmentation parameters were optimised for each data set. Curation of segmentation and tracking was carried out using BACMMAN’s interactive graphical interface. Although automated segmentation and tracking was mostly accurate, occasionally errors were produced. Thus, every lineage was manually checked, with 2‐pal data sets requiring the most curation mainly due to excessive elongation of cells expressing the SOS response. Mother cells that did not grow for the duration of the experiment and those that were already excessively elongated at the beginning of the experiment were removed. Positions with channel deformities and where loss of focus occurred were also discarded. Cell fluorescence and morphology variables were then exported from BACMMAN into excel files for further processing.

Further analysis was performed using custom MATLAB scripts. First, data corresponding to the “mother cell” lineage were isolated. Divisions for the mother cell lineage were recorded with tracking continuing for the cell at the closed end of the channel and discarding the sister cell. Division rates were calculated as the inverse of the interdivision time. The population growth rate was then estimated from the division rates as explained in the supplementary material. Cell elongation rates were calculated using a linear fit to the logarithm of cell length as a function of time per cell cycle. Cell length was determined as the maximal distance between two points of the cell contour as reported by BACMMAN. A minimum of 3 data points per generation was imposed as a fitting constraint and negative growth rates were removed. The rate of reaching a high SOS state (α) was estimated based on GFP fluorescent value as described in the Appendix Supplementary Methods and Appendix Fig S11.

## Author contributions

**Sebastián Jaramillo‐Riveri:** Conceptualization; Software; Formal analysis; Validation; Investigation; Visualization; Methodology; Writing—original draft; Writing—review & editing; co‐first author. **James Broughton:** Conceptualization; Software; Formal analysis; Investigation; Visualization; Methodology; Writing—review & editing; co‐ first author. **Alexander McVey:** Resources; Methodology. **Teuta Pilizota:** Conceptualization; Resources; Methodology. **Matthew Scott:** Conceptualization; Formal analysis; Investigation; Writing—original draft; Writing—review & editing. **Meriem El Karoui:** Conceptualization; Formal analysis; Supervision; Funding acquisition; Investigation; Methodology; Writing—original draft; Project administration; Writing—review & editing.

In addition to the CRediT author contributions listed above, the contributions in detail are:

SJ‐R, MS and MEK conceived the experiments. SJ‐R and JB performed the experiments and contributed equally to the work. AMV, SJ‐R, TP and MEK developed and AMV and SJ‐R designed microfluidic technology. SJ‐R, JB and MEK analyzed the data. SJ‐R, JB, MS and MEK interpreted the data and wrote the manuscript. All authors reviewed the manuscript.

## Disclosure and competing interests statement

The authors declare that they have no conflict of interest.

## Supporting information



Appendix

Expanded View Figures PDF

Dataset EV1

Dataset EV2

## Data Availability

The data sets and computer code produced in this study are available at the following locations:
Microscopy images: BioImage Archive, https://www.ebi.ac.uk/biostudies/BioImages/studies/S‐BSST833 and University of Edinburgh DataShare (https://doi.org/10.7488/ds/3064). The models and algorithms developed in this study are described in the Appendix. Custom scripts used for data analysis, cell segmentation (agar‐pad datasets), microscope control, and designs for the mother machine fabrication masks are available on GitLab: https://gitlab.com/MEKlab/growth‐sos‐msb‐2021. The parameters estimated from image analysis and used as parameters for the models are summarized in Dataset EV1 (snapshot data) and Dataset EV2 (Mother Machine data).
