# Supplementary material for: Growth‐dependent heterogeneity in the DNA damage response in Escherichia coli
Source: Mol Syst Biol. 2022 May 27;18(5):e10441. doi: 10.15252/msb.202110441 (PMC9136515; doi:10.15252/msb.202110441)
Supplement: Supplementary file 1 — Appendix [file MSB-18-e10441-s001.pdf]

Appendix

April 6, 2022

Contents

1 Supplementary Figures and Tables

2 Supplementary Methods
2.1 List of plasmids, strains, and primers
2.2 Image analysis of snapshot data
2.3 Mother machine
2.3.1 Microfluidics device fabrication
2.3.2 Mother machine data analysis
2.4 Estimating the transition rate to the high SOS state from mother machine experiments
2.5 Estimating the steady-state population growth-rate from single-cell division time distributions
2.6 Estimating the fraction of high SOS cell in a growing population

1 Supplementary Figures and Tables

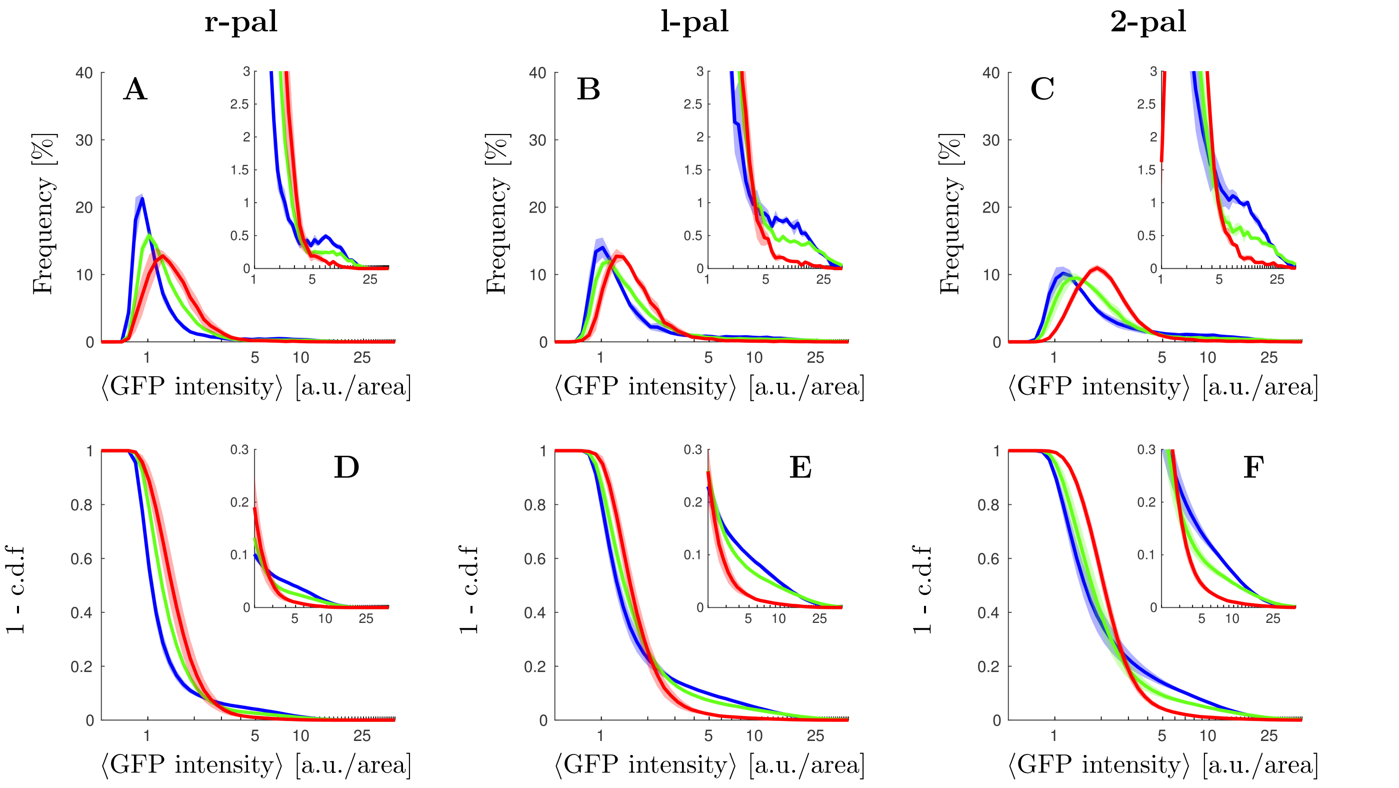

Appendix Figure S1: Single cell distributions of GFP intensity under replication-dependent DSBs For all plots, growth conditions are: M9-glycerol (blue), M9-glucose (green), and M9-glucose+amino-acids (red). Solid lines represent the average and shaded area the standard error from at least 3 replicates done in different days.

A) Steady state distribution of GFP intensity from SOS reporter *PsulA-mGFP* for cells under replication-dependent DSBs from the r-pal palindrome, in different growth conditions.

B) Steady state distribution of GFP intensity from SOS reporter *PsulA-mGFP* for cells under replication-dependent DSBs from the l-pal palindrome, in different growth conditions.

C) Steady state distribution of GFP intensity from SOS reporter *PsulA-mGFP* for cells under replication-dependent DSBs from both l-pal and r-pal palindromes (2-pal), in different growth conditions. Figure panel reused from Figure 2A for comparison purposes.

D) Steady state cumulative distribution of GFP intensity from SOS reporter *PsulA-mGFP* for cells under replication-dependent DSBs from the r-pal palindrome, in different growth conditions.

E) Steady state cumulative distribution of GFP intensity from SOS reporter *PsulA-mGFP* for cells under replication-dependent DSBs from the l-pal palindrome, in different growth conditions.

F) Steady state cumulative distribution of GFP intensity from SOS reporter *PsulA-mGFP* for cells under replication-dependent DSBs from both l-pal and r-pal palindromes (2-pal), in different growth conditions.

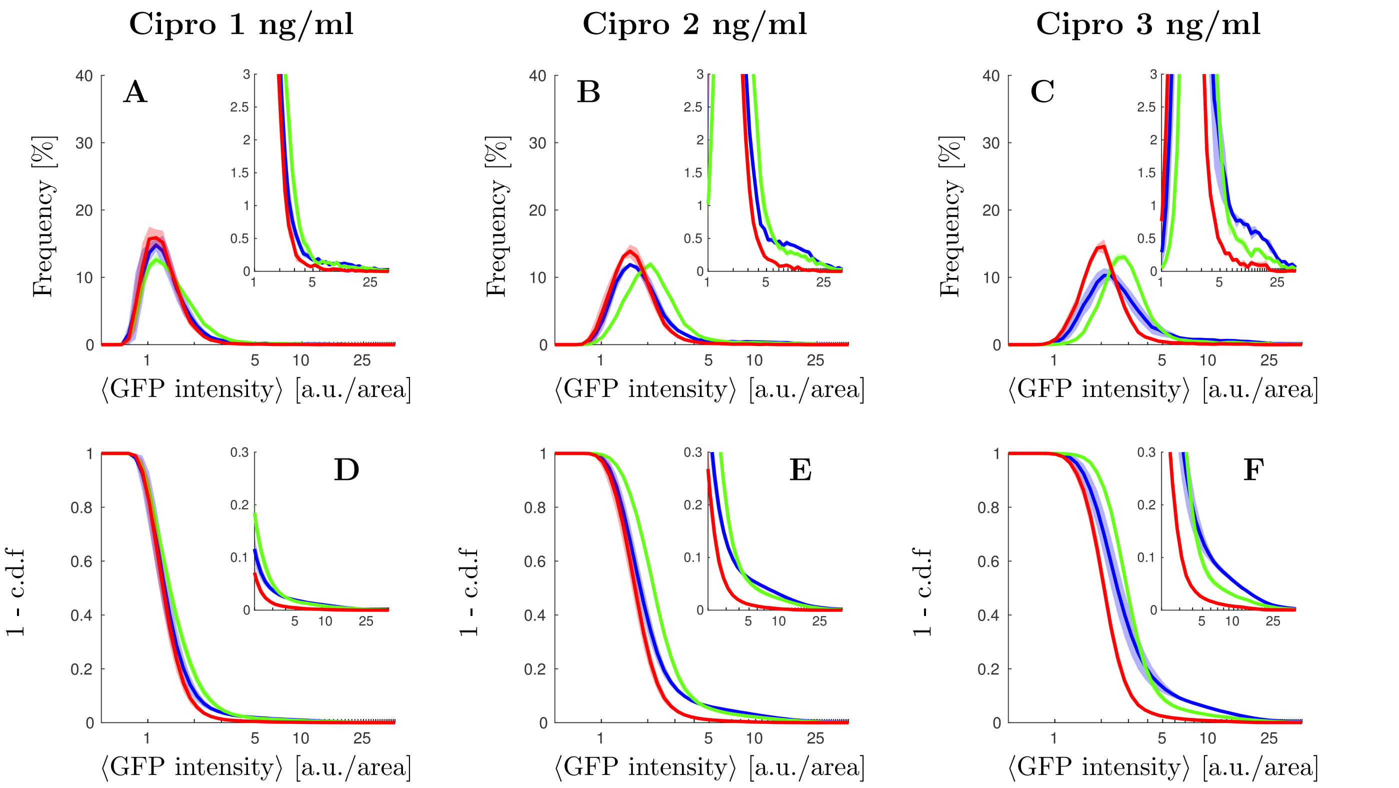

Appendix Figure S2: Single cell distributions of GFP intensity under ciprofloxacin

For all plots, growth conditions are: M9-glycerol (blue), M9-glucose (green), and M9-glucose+amino-acids (red). Solid lines represent the average and shaded area the standard error from at least 3 replicates done in different days.

A,B,C) Steady state distribution of GFP intensity from SOS reporter *PsulA-mGFP* for cells exposed to 1 ng/ml (respectively 2 ng/ml, 3 ng/ml) of ciprofloxacin in different growth conditions.

D,E,F) Steady state cumulative distribution of GFP intensity from SOS reporter *PsulA-mGFP* for cells exposed to 1 ng/ml (respectively 2 ng/ml, 3 ng/ml) of ciprofloxacin in different growth conditions. Figure panel F reused from Figure 2B for comparison purposes.

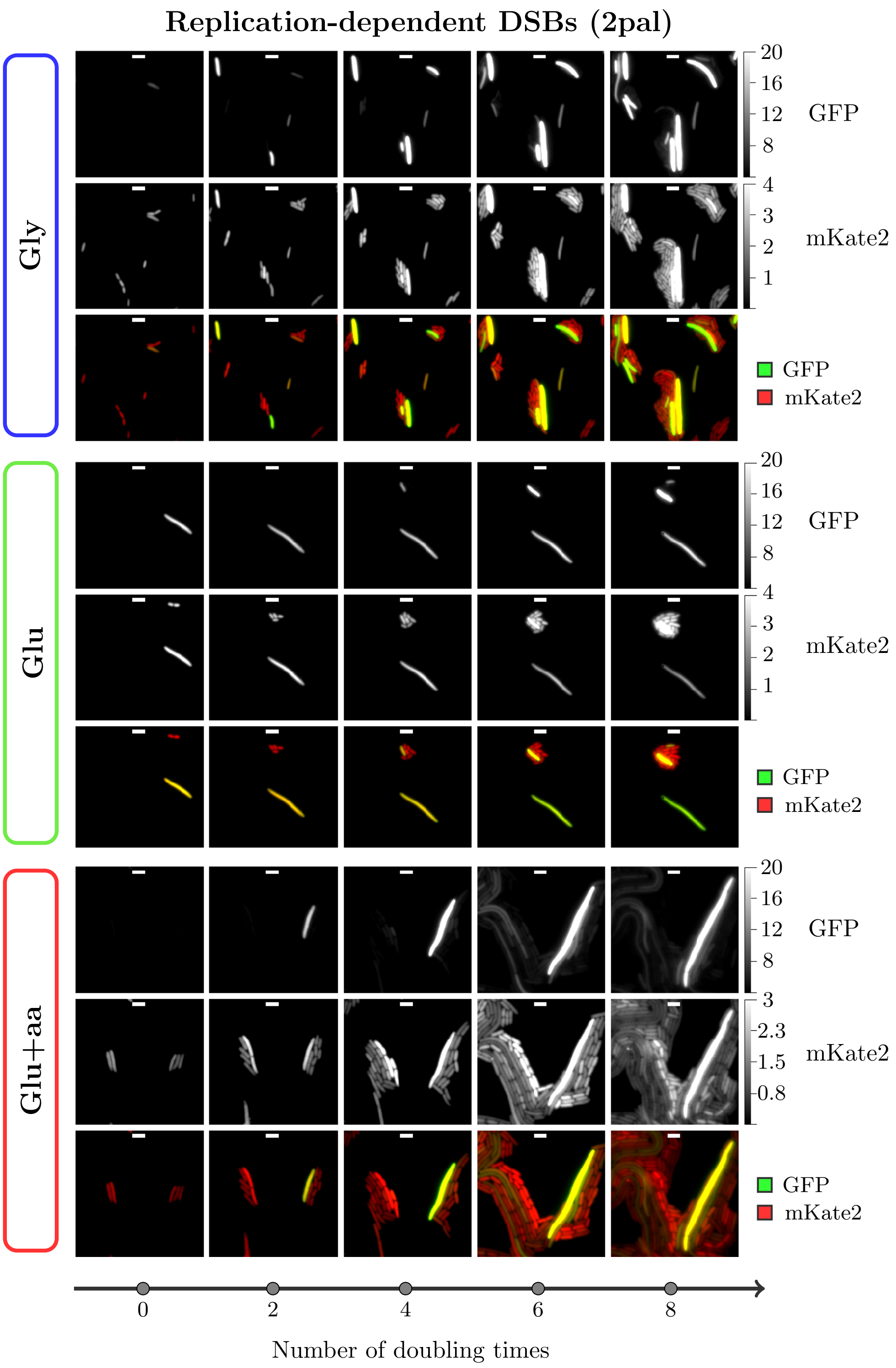

Appendix Figure S3: Time-lapse microscopy of cells under replication-dependent DSBs
Cells carrying two palindromes, the SOS reporter *PsulA-mGFP*, and a constitutively expressed reporter *PtetO-mKate2*, were imaged using agar-pads made with different growth media. Cells were grown to steady-state exponential growth before mounting into the agar-pads.

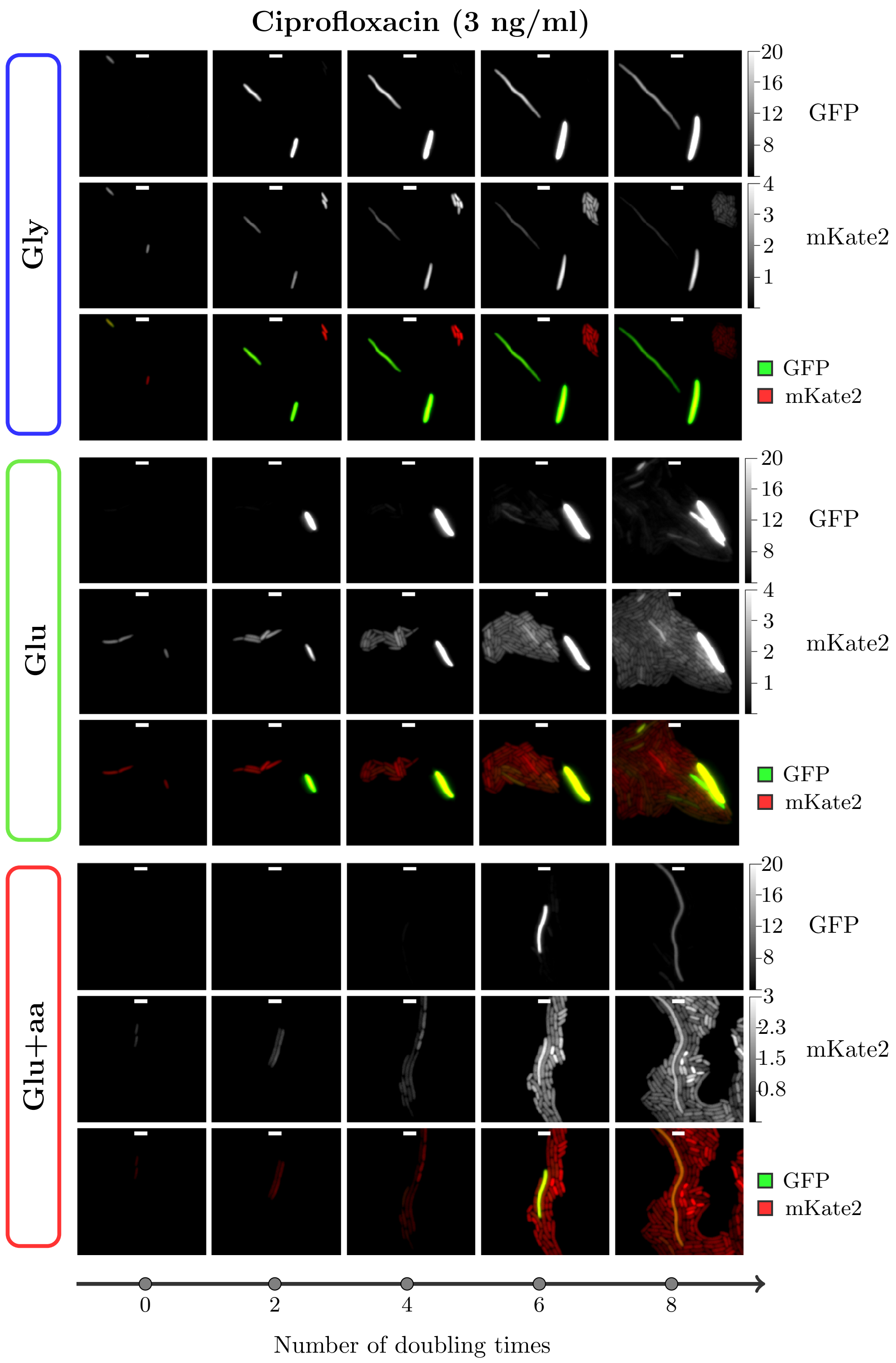

Appendix Figure S4: Time-lapse microscopy of cells exposed to ciprofloxacin
Cells carrying the SOS reporter *PsulA-mGFP*, and a constitutively expressed reporter *PtetO-mKate2*, were imaged using agar-pads made with different growth media supplemented with 3 ng/ml of ciprofloxacin. Cells were grown to steady-state exponential growth in exposure to the antibiotic before mounting into the agar-pads.

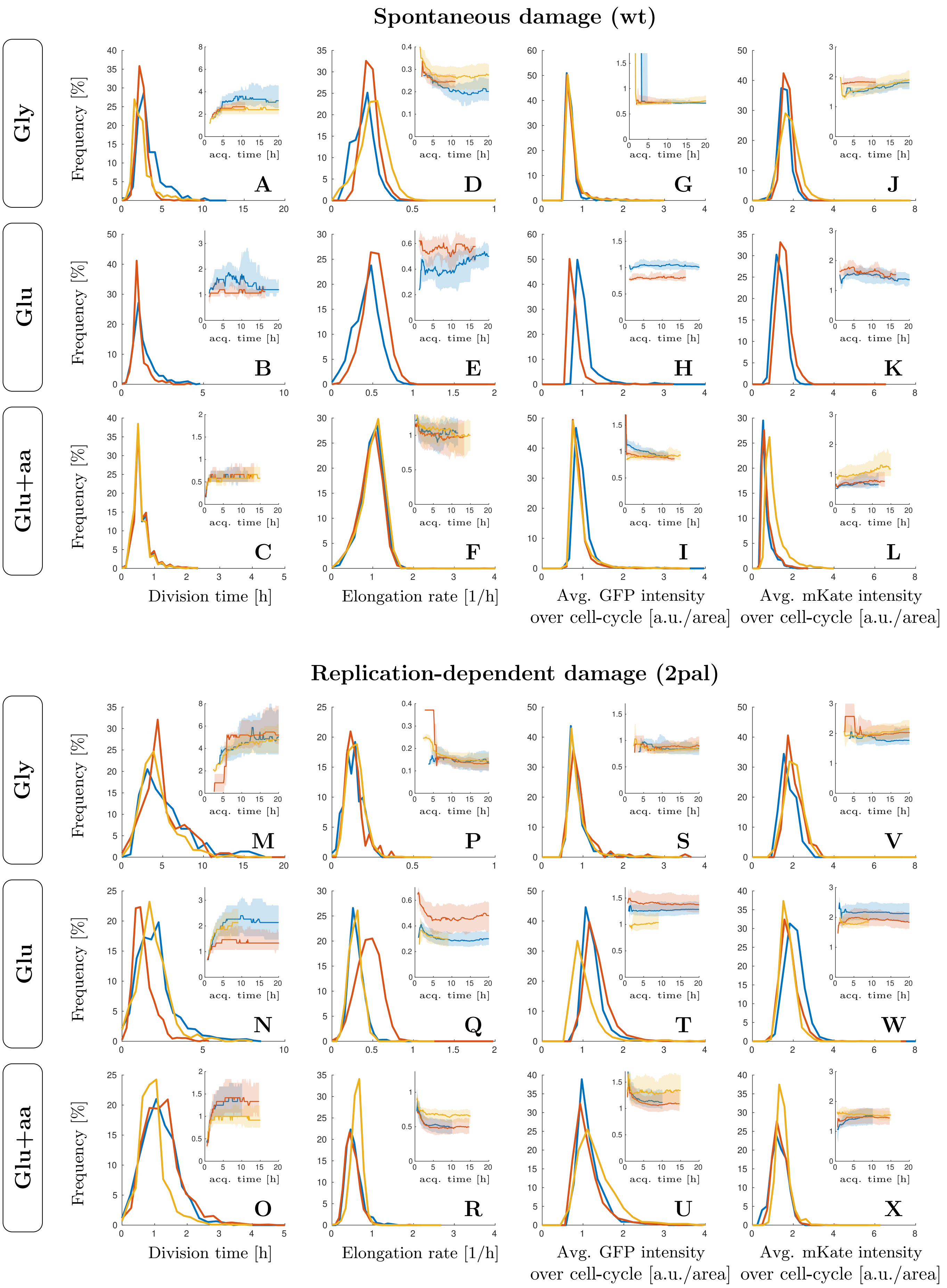

Appendix Figure S5: Distribution of main single-cell parameters from mother-machine experiments The lineages of wild-type cells and cells undergoing replication dependent DNA-damage were tracked for each cell cycle, and their division time, elongation rate, average fluorescence intensity in the GFP and mKate2 channels recorded. The

distribution of these 4 values are presented. Each color (Red, yellow and blue) represents an independent biological repeat. On each panel inset, we show the median values over time, and with shaded areas representing the first and third quartile of each distribution. For each timepoint in the inset curves, only cell-cycles spanning that particular time-point were included for computations of the median, first and second quartiles.

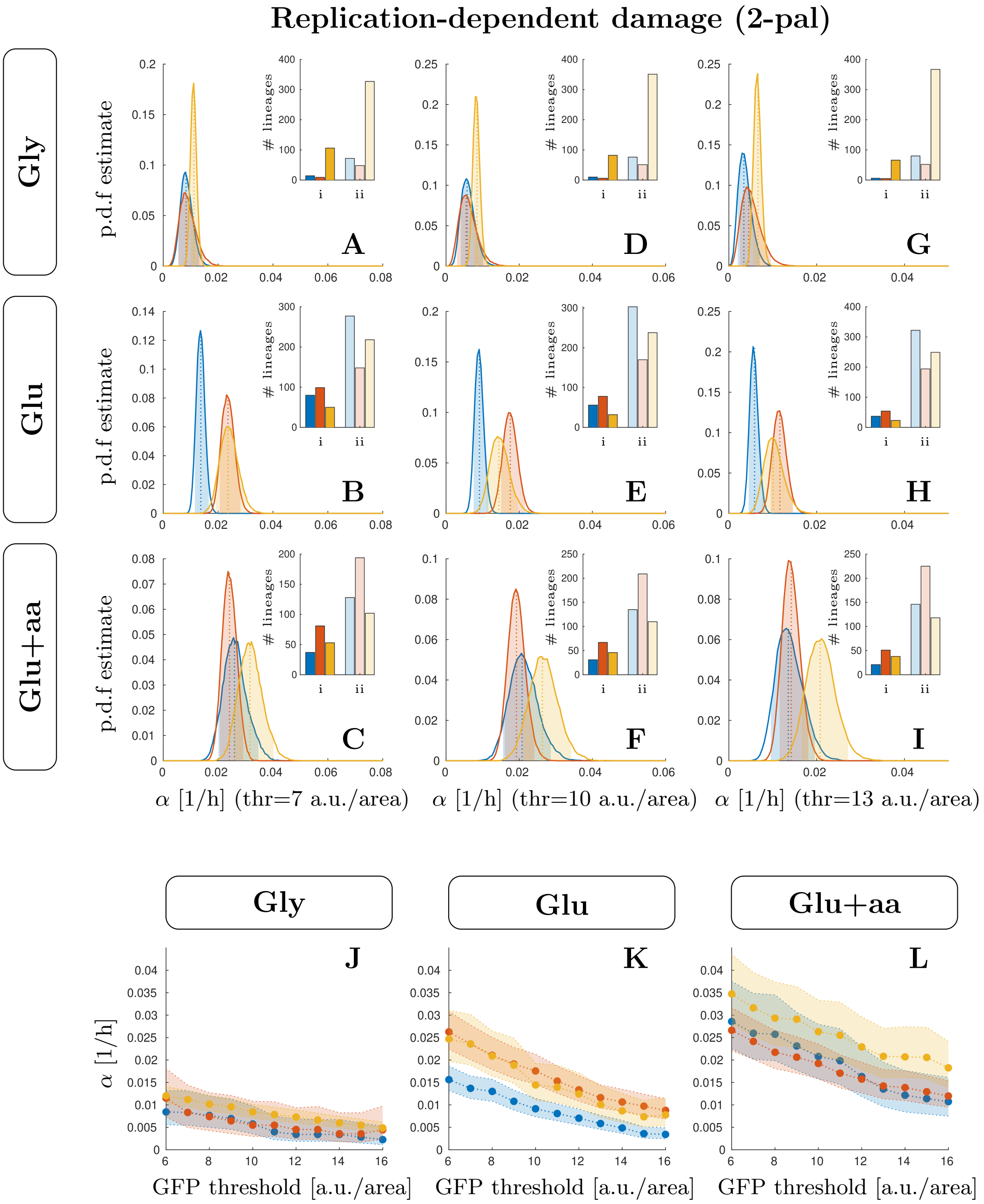

Appendix Figure S6: Inference of the transition rate to high SOS from individual cell traces at different GFP intensity thresholds

The single cell GFP intensity trajectories were used to estimate the transition rate constant (α) based on different GFP intensity threshold. The posterior probability of based on the observations was computed from dividing lineages into those that cross and do no cross the threshold, those are case "i" and "ii" respectively. Examples of these posterior probabilities are presented in panels A-I, where the shared areas represent the range between the 5th and 95th percentiles. The inlets in panels A-I are used to show the number of lineages on each category. In panels J-L we show the most likely value of α for the different growth-conditions, and shaded areas represent the 5th and 95th percentiles from the posterior distributions. In all panels, the color red, yellow, and blue are used to represent three independent biological repeats.

Appendix Table S1: Comparison of doubling rates estimated from batch growth curves (OD600 absorbance, CFUs) and single-cell (mother machine) data for WT and 2-pal strains under different growth conditions.

Strain: WT

**Growth Medium**	**OD600 [dbl/hr]**	**CFUs [dbl/hr]**	**Mother-machine [dbl/hr]**
M9-glucose+amino-acids	1.61	1.46	1.76
M9-glucose	1.04	0.95	0.85
M9-glycerol	0.60	0.58	0.37

Strain: 2-pal

**Growth Medium**	**OD600 [dbl/hr]**	**CFUs [dbl/hr]**	**Mother-machine [dbl/hr]**
M9-glucose+amino-acids	1.42	1.08	0.92
M9-glucose	0.81	0.72	0.61
M9-glycerol	0.45	0.33	0.21

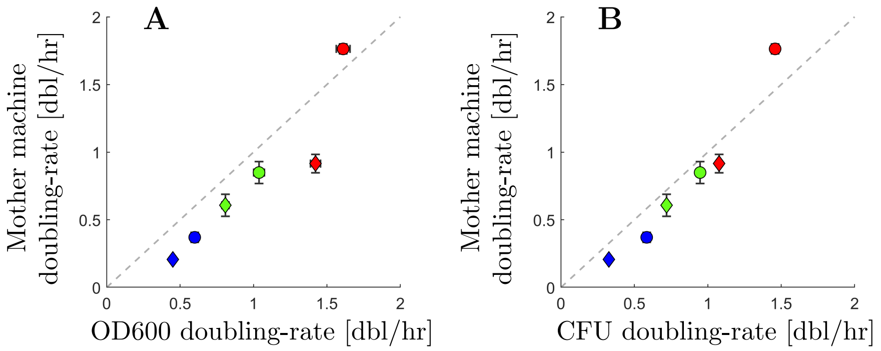

Appendix Figure S7: Comparison of doubling rates estimated from single-cell mother machine experiments and batch growth experiments

Comparison between doubling rates for the WT (circles) and 2-pal mutant (diamonds) estimated from growth in the mother machine and in batch in different growth conditions. Mother machine doubling rates are the estimated population doubling rate derived from the underlying single-cell division-rate distribution. Points represent the average and bars the standard error from biological repeats.

A) Batch doubling-rates estimated from OD600 growth curves compared to mother machine doubling-rates. Figure panel reused from Figure 4C for comparison purposes.

B) Batch doubling-rates estimated from CFU/ml (Colony Forming Units) growth curves compared to mother machine doubling-rates.

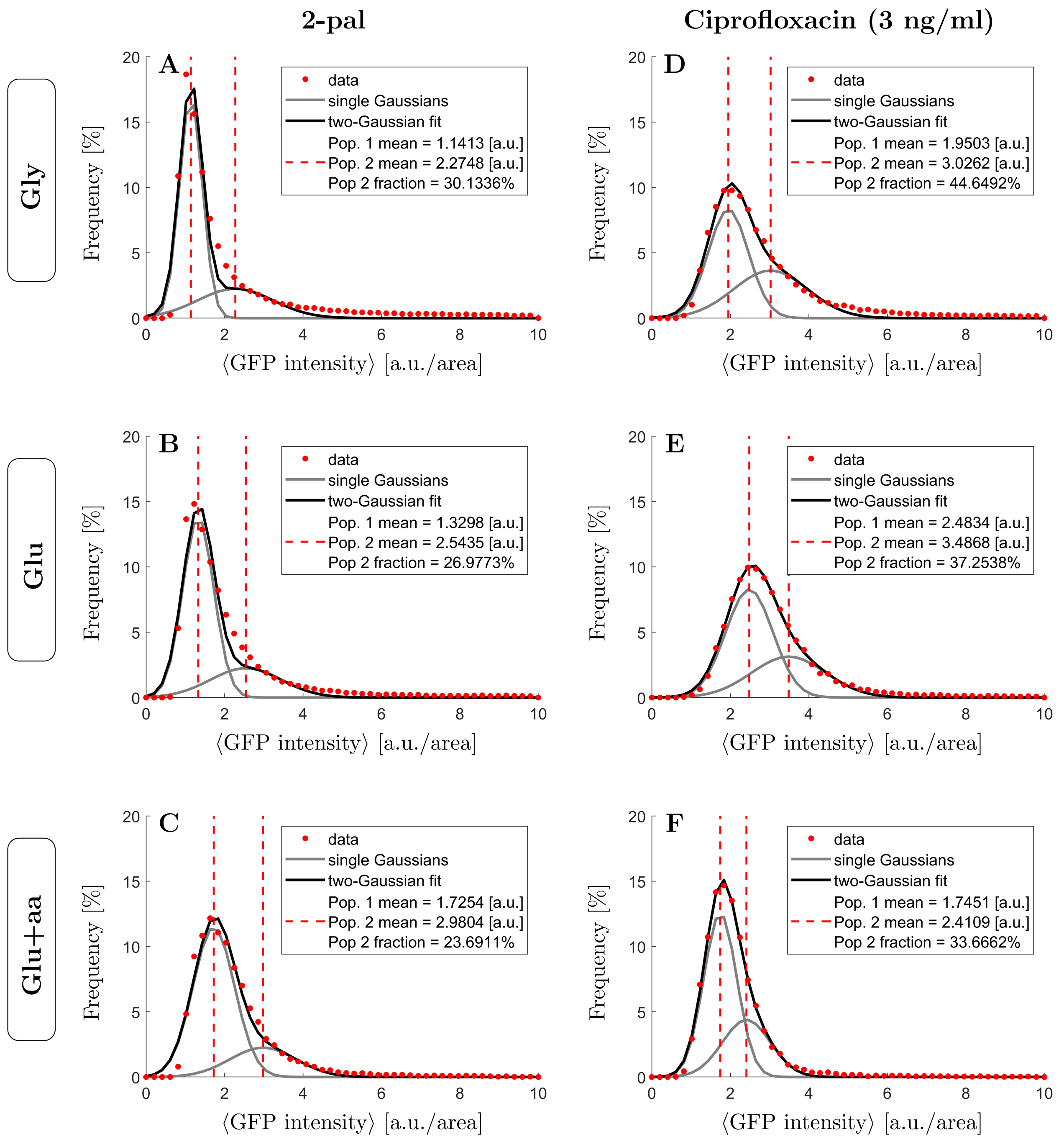

Appendix Figure S8: Two-population peak-fitting to single-cell steady-state distributions of GFP intensity under replication-dependent DNA damage and ciprofloxacin

For all plots, growth conditions are: M9-glycerol (Gly), M9-glucose (Glu), and M9-glucose+amino-acids (Glu+aa). Red dots represent the value of GFP intensity averaged from at least 3 replicates done on different days. Solid black lines represent the fitting of a two-Gaussian model to the data. Solid grey lines are the two underlying Gaussians obtained from the two-Gaussian fit. Also indicated is the mean GFP intensity estimated for the two populations (dashed red lines), and the fraction of population 2.

A,B,C) Steady-state distribution of GFP intensity from SOS reporter *PsulA-mGFP* for cells under replication-dependent DNA damage in different growth conditions.

D,E,F) Steady-state distribution of GFP intensity from SOS reporter *PsulA-mGFP* for cells exposed to 3 ng/ml of ciprofloxacin in different growth conditions.

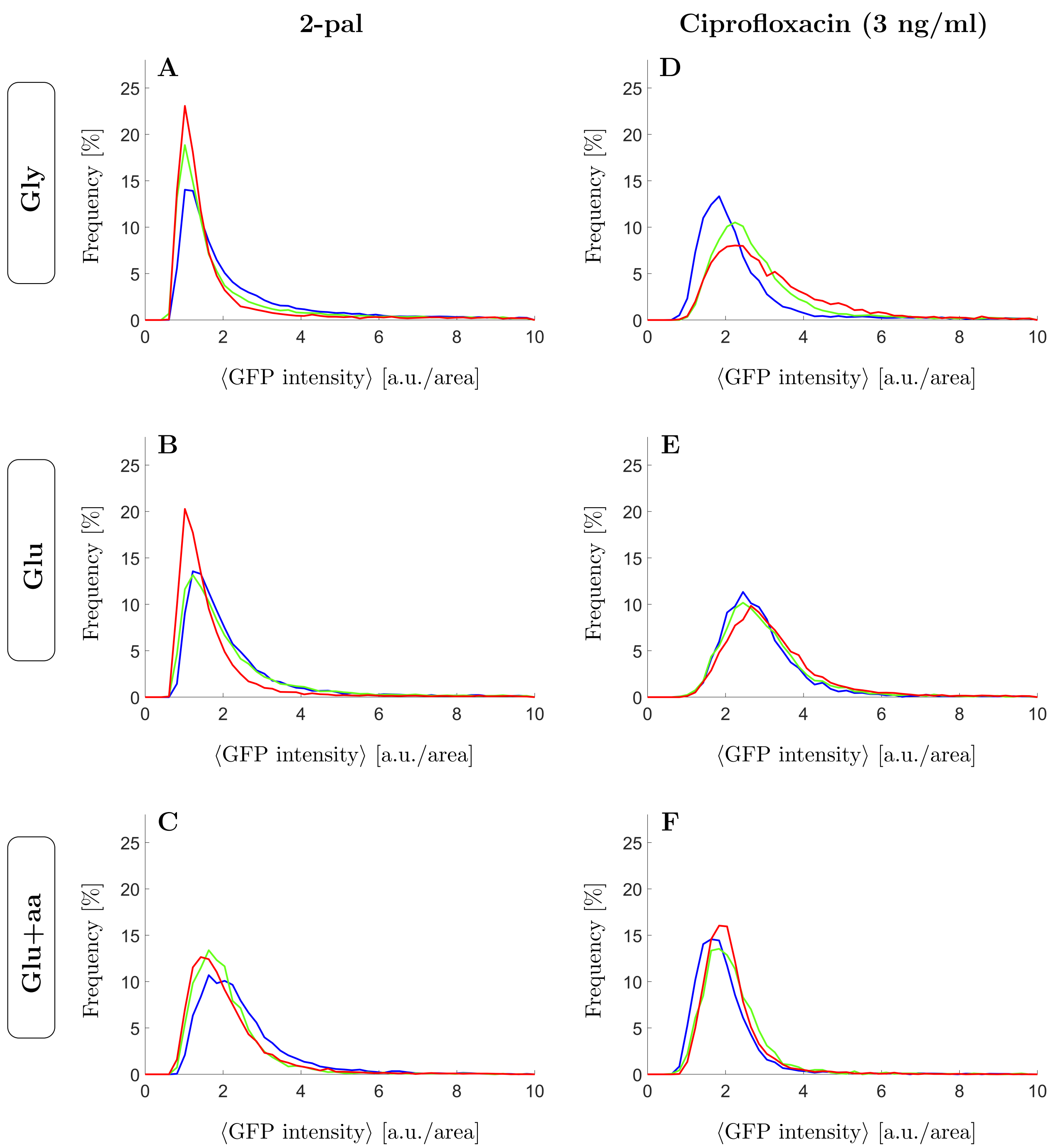

Appendix Figure S9: Single-cell steady-state distributions of GFP intensity under replication-dependent DNA damage and 3 ng/ml ciprofloxacin

For all plots, growth conditions are: M9-glycerol (Gly), M9-glucose (Glu), and M9-glucose+amino-acids (Glu+aa). Different colours represent experimental repeats done on different days.

A,B,C) Steady-state distribution of GFP intensity from SOS reporter *PsulA-mGFP* for cells under replication-dependent DNA damage in different growth conditions.

D,E,F) Steady-state distribution of GFP intensity from SOS reporter *PsulA-mGFP* for cells exposed to 3 ng/ml of ciprofloxacin in different growth conditions.

2 Supplementary Methods

2.1 List of plasmids, strains, and primers

Here are the list of strains, plasmids and primers used in this study. Bacterial strains were constructed either by P1 transduction or by clone-integration (St-Pierre et al., 2013) as mentioned in Table S2. Plasmids pSJR036 and pSJR046 were constructed by digestion of the backbone by enzymatic restriction, amplification of the insert by PCR using a high fidelity polymerase and ligation using Gibson assembly (details in Table S4). All plasmids were checked by PCR amplification of the insert and Sanger sequencing. After construction, all strains were checked by PCR amplification and Sanger sequencing of the modified chromosomal region.

Appendix Table S2: List of strains. CLI stands for clone-integration, and P1 for phage transduction.

Strain	Background	Genotype	Source/Construction
eSJR017	MG1655	*seqA::mGFP* P_{rna1}-*mKate2*	Gift from Raul Fernandez Lopez (RFL84). mKate2 construct built by Nathan Lord.
eSJR048	MG1655	*rph-1* λ^-	Genomic Stock Center (CGSC7740)
eSJR059	MG1655	*lacI^q^ lacZ::pal246 cynX::Gm*^{R}	Gift from David Leach (DL2859)
eSJR130	BW27784	*asbB::pal246 ascF::Kn*^{R}	Gift from David Leach (DL4212)
eSJR145	MG1655	*HK022*:P_{sulA}-*mGFP*	eSJR048 CLI using pSJR036
eSJR206	MG1655	*HK022*:P_{sulA}-*mGFP* *P21*:P_{tet01}-*mKate2*	eSJR145 CLI using pSJR046
eSJR214	MG1655	eSJR206 *asbB::pal246 ascF::Kn*^{R}	eSJR206 P1 using eSJR130
eSJR301	MG1655	eSJR206 *lacI*^{q} *lacZ::pal246 cynX::Gm*^{R}	eSJR206 P1 using eSJR059
eSJR302	MG1655	eSJR206 *lacI*^{q} *lacZ::pal246 cynX::Gm*^{R} *asbB::pal246 ascF::Kn*^{R}	eSJR301 P1 using eSJR130

Appendix Table S3: List of plasmids.

Plasmid	Purpose	Source
pSJR017	Clone-integration marker excision	pE-FLP (St-Pierre et al., 2013)
pSJR021	Clone-integration at HK022 site	pOSIP-KH (St-Pierre et al., 2013)
pSJR025	Clone-integration at P21 site	pOSIP-KT (St-Pierre et al., 2013)
pSJR035	Source sequence for P*sulA-mGFP*	DL4847
pSJR036	P_{sulA}-*mGFP* insertion by CLI	This study
pSJR046	P_{tetO1}-*mKate2* insertion by CLI	This study

Appendix Table S4: Plasmid construction. List of plasmids constructed in this study.

Plasmid		Backbone	Digestion	PCR template(s)	primer pair(s)
pSJR036	*HK022::*P_{sulA}-*mGFP*	pSJR021	EcoRI, PstI	pSJR035	oSJR084, oSJR085
pSJR046	*P21::*P_{tetO1}-*mKate2*	pSJR025	EcoRI, PstI	eSJR017	oSJR066, oSJR098

Appendix Table S5: List of primers.

Primer	5′-3′ Sequence	Purpose
oSJR084	GGACGCCCGCCATAAACTGCCAGGAATTGG<br>GGATCGGAATTCAGGGTTGATCTTTGTTGT	pSJR036 construction
oSJR085	TTAGGTTAGGCGCCATGCATCTCGAGGCAT<br>GCCTGCAGTTATTTGTATAGTTCATCCATG	pSJR036 construction
oSJR066	ACGCCCGCCATAAACTGCCAGGAATTGGGG<br>ATCGGAATTCTTATCTGTGCCCCAGTTTGC	pSJR046 construction
oSJR098	ATGAATTCAAATACTGTCCTTCCGGTCAGT<br>GCGTCCTGCTGATGTGCTCAGTATCTCTAT<br>CACTGATAGGGATGTCAATCTCTATCACTG<br>ATAGGGACTCGACTGCAGGCATGCCTCGAG<br>ATGCATGGCGCCTAACCTAAACTGACA	pSJR046 construction
oSJR058	GGAATCAATGCCTGAGTG	HK022 insertion verification
oSJR059	ACTTAACGGCTGACATGG	HK022 insertion verification
oSJR060	ACGAGTATCGAGATGGCA	HK022 insertion verification
oSJR061	GGCATCAACAGCACATTC	HK022 insertion verification
oSJR092	ATCGCCTGTATGAACCTG	P21 insertion verification
oSJR093	ACTTAACGGCTGACATGG	P21 insertion verification
oSJR094	GGGAATTAATTCTTGAAGACG	P21 insertion verification
oSJR095	TAGAACTACCACCTGACC	P21 insertion verification
oSJR072	TTATGCTTCCGGCTCGTATG	*lacZ::pal246* verification FW
oSJR073	GGCGATTAAGTTGGGTAACG	*lacZ::pal246* verification RV
oSJR080	CCAACCAGTCTGAAGGTGCG	*ascB::pal246* verification FW
oSJR081	CCAGCGGTTCGATACCGTAC	*ascB::pal246* verification RV

2.2 Image analysis of snapshot data

In order to automate the detection of cells from fluorescent images (cell segmentation), we developed a custom algorithm based on edge-detection using low-pass filters (detailed in Algorithm 1) and a graphical user interface to facilitate manual correction of the segmentation. The algorithm was designed to detect cell edges using a custom convolution filter, that compares each pixel value relative to its neighbours. The filters are constructed by summing many 2D Gaussian distributions, where each Gaussian has a mean position moving away from the center of the filter with a given orientation (detailed in Algorithm 2). We apply several filters with different orientations to the fluorescence image, and compute a score by combining the results from all filters (Algorithm 1). Then a threshold is applied to the score to remove cell edges, and generate a mask from which cells are identified as individual connected components (see example in Figure S10. Finally, the resulting segmentation is manually curated to remove any potential misidentified cell.

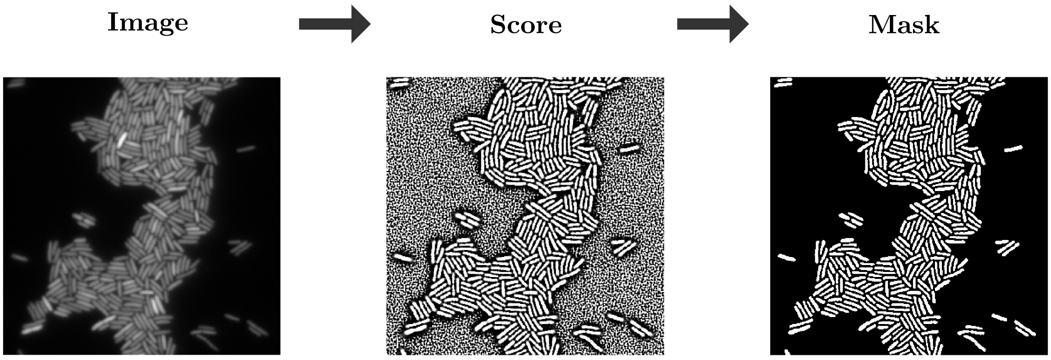

Appendix Figure S10: Semi-automated cell detection. Example of computed score and mask for a given image.

Algorithm 1: Cell segmentation from fluorescence image.

Algorithm for segmenting a fluorescent image using an array of spacial low-pass filters. It takes any image as input, plus seven parameters, and returns a mask containing where regions that appears as "valleys" in the intensity landscape have been removed

Require: Input image: img. Parameters: minimum intensity value i_0; μ and σ (gaussian filter); pixel length d, width w, and set of angles $A = \{A_1, A_1 + \pi\}$ (for low pass filter); and a score threshold s_0. Some predefined functions: IMFILTER that applies a convolution filter to an image; GAUSSFILTER that return a gaussian filter; THRESHOLD that thresholds an image returning a boolean matrix; LOWPASSFILTERS that computes custom low-pass filters (see algorithm 2); IMCOMPLEMENT that computes the complement of an image; POSBOOL that returns one if the value is positive; PAIRWMULT that computes the pairwise multiplication of matrices; and PAIRWDIV that computes the pairwise division of matrices.

---

function SEGMENTATION_MASK($img, i_0, \mu, \sigma, d, w, A, s_0$) ▷ Returns *mask*
  $img \leftarrow$ IMFILTER(GAUSSFILTER(μ, σ), img) ▷ Filter image noise
  $mask0 \leftarrow$ THRESHOLD(img, i_0) ▷ Threshold image
  $Filts \leftarrow$ LOWPASSFILTERS(d, w, A) ▷ Set low pass filters
  $na \leftarrow$ LENGTH(A) ▷ Number of filters
  for $j \leftarrow 1$ to na **do**
    $Fimg_j \leftarrow$ IMFILTER($Filts_j, img$) ▷ Set of filtered images
  end for
  for $j \leftarrow 1$ to $na/2$ **do**
    $Himg_j \leftarrow Fimg_j + Fimg_{j+na/2}$ ▷ Sum opposite angles
  end for
  $S_+ \leftarrow \sum_1^{na/2}$ PAIRWMULT($Himg_j$, POSBOOL($Himg_j$)) ▷ positives sum
  $S_- \leftarrow \sum_1^{na/2}$ PAIRWMULT($Himg_j$, $1 -$ POSBOOL($Himg_j$)) ▷ negatives sum
  $S_r \leftarrow$ PAIRWDIV($S_+, (S_- + 1)$) ▷ Compute ratios
  $S_l \leftarrow$ LOG($1 - S_r$) ▷ Take the log
  $score \leftarrow$ EXP(IMCOMPLEMENT(S_l)) ▷ Compute score
  $mask \leftarrow$ PAIRWMULT($mask0$, THRESHOLD($score, s_0$)) ▷ Final mask
  return *mask*
end function

Algorithm 2: lowpassfilters function.

Pseudocode for constructing an array of low pass filters. Each filter will compare each value relative to its neighbours, but only in an angle. Constructing the filter using a 2D Gaussian density function makes the filter less sensitive to image noise.

Require: Parameters: pixel length d, width w, and set of angles $A = \{A_1, A_1 + \pi\}$. Some predefined functions: GAUSSPROJ returning the integral of a 2D gaussian density function (with mean x, y and standard deviation w) over a space grid; and SUM2 that sums all elements of a matrix.

---

function LOWPASSFILTERS(d,w,A) ▷ Returns cell array $Filts$
    $na \leftarrow \mathrm{LENGTH}(A)$ ▷ Number of filters
    $ngrid \leftarrow 2d + 1$ ▷ Size of filter
    for $j \leftarrow 1$ to na **do** ▷ Compute filter for each angle
        $Filts_j \leftarrow \mathrm{ZEROS}(ngrid, ngrid)$ ▷ Initialise to zeros
        $a \leftarrow A(j)$ ▷ angle
        for $q \leftarrow 1$ to d **do** ▷ move from center to d
            $x \leftarrow q * \mathrm{COS}(a)$ ▷ X projection
            $x \leftarrow q * \mathrm{SIN}(a)$ ▷ Y projection
            $Filts_j \leftarrow Filts_j + \mathrm{GAUSSPROJ}(x, y, w)$ ▷ Accumulate 2D distributions
        end for
        $\theta \leftarrow \mathrm{SUM2}(Filts_j)$ ▷ Sum all values so far
        $Filts_j \leftarrow \theta * \mathrm{GAUSSPROJ}(0, 0, w) - Filts_j$ ▷ Final local difference filter
    end for
    return $Filts$
end function

2.3 Mother machine

2.3.1 Microfluidics device fabrication

The protocol used to fabricate the microfluidics chips is summarised as follows: First, the surface of the master wafer was treated with silane to facilitate removal of PDMS from the surface. The wafer was then taped to the bottom of a large petri dish to secure it in place. Mixed PDMS (1:10 ratio of curing agent to base) was poured onto the master mould to achieve a thickness of approximately 5 mm. The freshly poured PDMS was degassed for 1 h in a vacuum bell jar to remove bubbles followed by curing at 65°C overnight. After cooling to room temperature, chips were then carefully cut out using a scalpel and feeding channels created using a 0.75 mm (ID) biopsy punch (World Precision Instruments Limited). Chips were cleaned by sonicating in isopropanol for 30 min and then left to air dry overnight at 65°C in a closed petri dish (features facing up). Coverslips (Duran, 24x60 mm, #1.5) were cleaned by sonicating in 1 M KOH for 30 min, followed by rinsing three times in Milli-Q water, and then sonication for a further 30 min in Milli-Q water. Coverslips were left to dry overnight at 65°C. Before bonding, chips and coverslips were surface-activated in an oxygen plasma cleaner operated at high intensity under vacuum for 60 s. Bonded chips were then left at 65°C for at least 10 min followed by storage in parafilm-sealed petri dishes at room temperature.

Appendix Table S6: Microchannel dimensions used for different growth media.

Growth Medium	Height (μm)	Width (μm)	Length (μm)
M9-glucose+amino-acids	1.36±0.08	1.4-1.6	26
M9-glucose	0.91±0.03	1.1-1.3	25±2
M9-glycerol	0.91±0.03	1.1-1.2	25±2

2.3.2 Mother machine data analysis

Additional notes on data curation for mother machine data sets: Initially cells had yet to fully adapt to the imaging conditions as indicated by the mkate2 signal degrading for approximately 2 hours after imaging began, after which it stabilised. This data was discarded and not used for data analysis. At most, 4 hours were truncated from the beginning of data sets. For growth rate and division rate calculations, all non-growing cell cycles (defined by a minimum growth rate of 0.03 h^{-1}) were discarded.

2.4 Estimating the transition rate to the high SOS state from mother machine experiments

To analyse the Mother Machine data we used a simple mathematical model (described in the main text). Cells can switch from low to high SOS at rate α and we assume switching back to low SOS is very rare so we neglect this possibility. We assume that this switching can be described by a Poisson process. We estimated α by Maximum likelihood in each nutrient condition using single-cell time-lapse recording of GFP intensity from the Mother Machine. Only mother cells were included in the analysis.

Assuming conversion from low to high SOS can be described by a Poisson process, the time it takes for a given lineage to pass some critical GFP intensity follows an exponential distribution with rate α. We call such elapsed time t_S. Then, for every lineage beginning at a low-SOS levels (i.e. GFP below the threshold) we distinguish two outcomes: i) the lineage crosses the threshold at a given time from the first observation, ii) the lineage does not cross the threshold over the whole period when it is observed. Given t_S is assumed to follow an exponential distribution, we can estimate the probabilities associated to each event class.

For the first case, we will describe its probability as the probability that t_S falls within the current and previous time interval. Whereas the probability associated to the second case is the probability that t_S is larger than the total observed time for that lineage (i.e. 1 minus the c.d.f of t_S). Then

Case	Description	Probability
i	Lineage crosses GFP theshold at time t	$\mathbb{P}(t - t_\Delta > t_S \geq t \vert \alpha) = e^{-\alpha t}\left(e^{\alpha t_\Delta} - 1\right)$
ii	Lineage does not crosses GFP theshold at time t	$\mathbb{P}(t_S > t \vert \alpha) = 1 - (1 - e^{-\alpha t}) = e^{-\alpha t}$

where t_Δ is the time interval used for imaging.

With these definitions, we classify each lineage into both cases, and define a likelihood for each set of observations. Let's call the set of all elapsed times for the lineages in the first case by T_1, and the total observed time for all lineages in the second case by T_2. Then we define the likelihood of our observation as the product of the probabilities of their respective cases

$$\mathcal{L} = \mathcal{L}_1\mathcal{L}_2 = \prod_{t_1 \in T_1} \mathbb{P}(t_1 - t_\Delta > t_S \geq t_2|\alpha) \prod_{t_2 \in T_2} \mathbb{P}(t_S > t_2|\alpha)$$

where the log-likelihood is

$$\ln \mathcal{L}(\alpha) = |T_1| \ln \left(e^{\alpha t_\Delta} - 1\right) - \alpha \left(\sum_{t_1 \in T_1} t_1 + \sum_{t_2 \in T_2} t_2 \right) \tag{1}$$

where $|T_1|$ is the size of the set T_1, that is all observed cases that do cross the threshold. Also, using the partial derivative the log-likelihood we can obtain the value of α with maximum likelihood (α_m), that is

$$\alpha_m = \frac{1}{t_\Delta} \ln \left(\frac{\sum_{t_1 \in T_1} t_1 + \sum_{t_2 \in T_2} t_2}{\sum_{t_1 \in T_1} t_1 + \sum_{t_2 \in T_2} t_2 - |T_1| t_\Delta} \right) \tag{2}$$

In order to estimate the confidence in the estimation of α, we uzed the Metropolis-Hastings algorithm starting form α_m. During each iteration of the Markov chain, candidates were generated by sampling $\alpha' = \alpha_{i-1} + \delta$ where the index i represent the previous iteration, and $\delta \sim uniform([-\epsilon, \epsilon])$. Whenever some sampled candidate α' was found to be negative, α' was sampled again until it was not longer so. The acceptance ratio was computed using the log-likelihood, and was equal to $\exp\left(\ln \mathcal{L}(\alpha') - \ln \mathcal{L}(\alpha_i)\right)$. Monte-Carlo simulations were performed through 10^5 iterations using $\epsilon = 10^{-3}$.

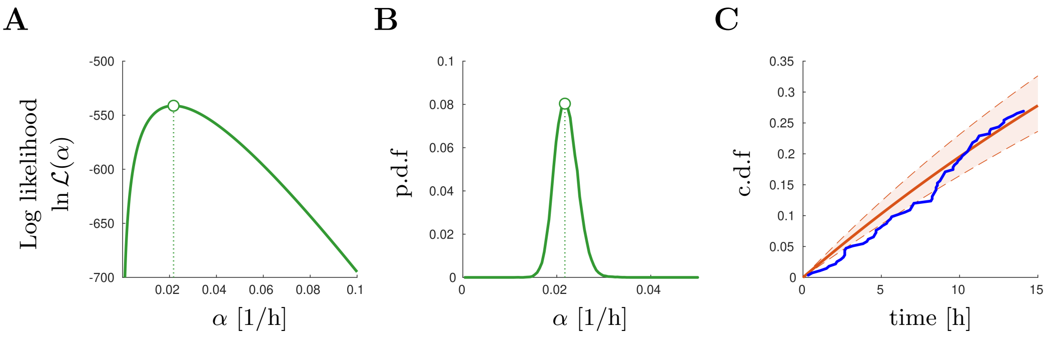

Appendix Figure S11: Estimation of the switching rate α from mother-machine experiments
Given a GFP intensity (SOS) threshold, we assume that the conversion between low and high SOS can be described by a Poisson process which allows us to derive explicit expressions for the likelihood of SOS transitions based on the transition rate α. In particular, we can obtain the value of α with maximum likelihood (panel A), and use Metropolis-Hastings algorithm to estimate the p.d.f of α (panel B). In panel C we compare the empirical cumulative density function of elapsed times to pass the given threshold (blue line), with the expected exponential distribution (red line). The green shared area represents the 90% confidence interval based on the p.d.f estimates of α. This example was constructed using a GFP threshold of 8 A.U. using one of the dataset for the strain carrying 2 palindromes in glucose + amino-acids condition.

2.5 Estimating the steady-state population growth-rate from single-cell division time distributions

Different division times distributions will result in populations "effectively" growing at different rates, and to compute this value taking the average division time is not accurate (Painter and Marr, 1968; Thomas, 2017). To avoid confusions, we wish to clarify that we are referring here specifically to the growth-rate of the population size in number of cells.

The relation between the distribution of division times and the population growth-rate is given by a functional equation and cannot be calculated explicitly (Painter and Marr, 1968; Thomas, 2017). Let's call τ a random variable denoting the division time which follows a $\phi(\tau)$ distribution and λ the steady-state population growth-rate. The population growth-rate λ satisfies the following equation assuming that correlation between successive division event can be neglected:

$$1 = 2 \int_0^\infty \phi(\tau)\ e^{-\lambda \tau} d\tau \tag{3}$$

In order to estimate λ from our experimental measurements, we follow a least-square strategy where λ is the value that minimizes the distance to the expected relation in equation 3. However, before we do this we need to estimate the distribution of division times $\phi(\tau)$ from our experimental measurements, taking into account that our measurements of division times are discrete due to the imaging protocol.

To simplify the problem, we divide $\phi(\tau)$ into separate intervals with width identical to the imaging acquisition interval (called t_Δ, and assume that the probability of observing a division event within that interval is uniform. We will use here an index $j \in \mathbb{N}_0^+$ to denote each interval. Finally, we make the simplification that the observed frequency of division events approximates the actual distribution. Then, combining these assumptions we have

$$\phi(\tau) \approx \frac{f(j)}{t_\Delta},$$

where $f(j)$ is the relative frequency of division events within an interval j. Note that for a given value of τ the corresponding interval will be different: $(j-1)t_\Delta < \tau \leq jt_\Delta$. Now we can combine our simplifications to replace the right side term of equation 3 and obtain

$$\int_0^\infty \phi(\tau)\; e^{-\lambda\tau} d\tau \;\approx \sum_{j=1}^{\infty} \frac{f(j)}{t_\Delta} \int_{(j-1)t_\Delta}^{jt_\Delta} e^{-\lambda\tau} d\tau = \frac{1}{\lambda t_\Delta} \left(e^{\lambda t_\Delta} - 1\right) \sum_{j=1}^{\infty} f(j) e^{-j\lambda t_\Delta}$$

Then, we estimate λ as the value that minimizes the following expression:

$$\lambda = \underset{\lambda \in \mathbb{R}^+}{\text{minimize}} \left(1 - 2\frac{1}{\lambda t_\Delta} \left(e^{\lambda t_\Delta} - 1\right) \sum_{j=1}^{\infty} f(j) e^{-j\lambda t_\Delta} \right)^2$$

Figure S12 shows that the minimal λ value is unique, and also reproducible between experimental repeats.

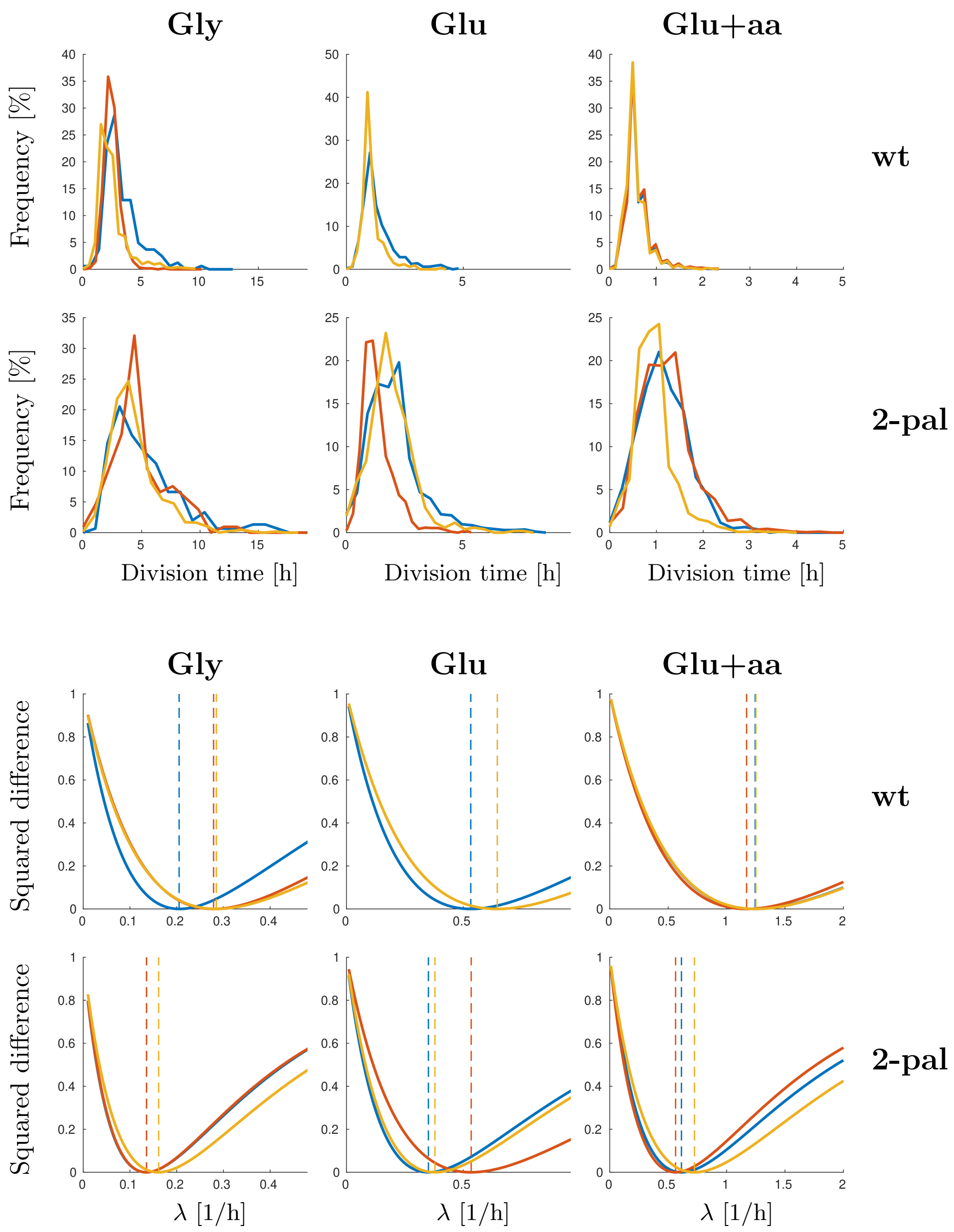

Appendix Figure S12: Estimation of population growth-rate from single-cell divisions
On the top, we display the division-time distributions for wild-type and 2-pal strains in three different media. On the bottom, we show the cost function associated to a given growth-rate value. The growth-rate with minimum value in the curve (dashed-line) corresponds to the estimated population growth-rate given the division time statistics. Each color represents one biological repeat.

2.6 Estimating the fraction of high SOS cell in a growing population

Let's consider n_1 and n_2 the number of cells in each state, and λ_1 and λ_2 the respective division rates. We call n_1 the number of "healthy" low SOS cells, and n_2 the number of slow-dividing cells with high SOS induction. Let us assume first-order kinetics for conversion between the two populations, and call α the conversion rate constant from population one to population two, and β the rate constant for the reverse reaction. Thus, the population dynamics is given by the following equations

$$\begin{aligned} \frac{dn_1}{dt} &= (\lambda_1 - \alpha)n_1 + \beta n_2 \\ \frac{dn_2}{dt} &= (\lambda_2 - \beta)n_2 + \alpha n_1 \end{aligned}$$

We are interested in the population fractions and call them $f_1 = \frac{n_1}{n_1+n_2} \in [0,1]$, and $f_2 = \frac{n_2}{n_1+n_2} \in [0,1]$. Converting the dynamics into fractions we get:

$$\begin{aligned} \frac{df_1}{dt} &= -\alpha f_1 + \beta f_2 + f_1 f_2(\lambda_1 - \lambda_2) \\ \frac{df_2}{dt} &= \alpha f_1 - \beta f_2 - f_1 f_2(\lambda_1 - \lambda_2) \end{aligned} \tag{4}$$

Notice that the total population growth is given by:

$$\frac{d(n_1+n_2)}{dt} = (\lambda_1 f_1 + \lambda_2 f_2)(n_1+n_2) \tag{5}$$

At steady-state, the population fractions are time-invariant. The solution for f_1 and f_2 at steady state is given by:

$$\begin{aligned} f_1 &= \frac{1}{2}\left(1 + \frac{\alpha+\beta\pm\sqrt{\delta}}{\lambda_2-\lambda_1}\right) \\ f_2 &= \frac{1}{2}\left(1 - \frac{\alpha+\beta\pm\sqrt{\delta}}{\lambda_2-\lambda_1}\right) \end{aligned} \tag{6}$$

where $\delta = (\beta + \alpha + \lambda_2 - \lambda_1)^2 - 4\beta(\lambda_2 - \lambda_1)$. The steady-state can be approximated when $\alpha \gg \beta$ (and therefore β is negligible as we assumed previously) and $\lambda_1 \gg \lambda_2$. Under this hypotheses we have:

$$\begin{aligned} f_2 &\approx \alpha/\lambda_1 \\ f_1 &\approx 1 - \alpha/\lambda_1 \end{aligned} \tag{7}$$

When λ_2 is not negligible (in slow growth condition where λ_1 is low), we get

$$f_2 \approx \alpha/\lambda_1 \left(\frac{1}{1 - \frac{\lambda_2}{\lambda_1}}\right) \tag{8}$$

Therefore, using this model, we can predict f_2, the fraction of high SOS cells based on the parameters measured in the Mother Machine and compare this prediction to the experimentally measured fraction of high SOS cells (see main text).

References

Painter, P. R. and Marr, A. G. (1968). Mathematics of microbial populations. *Annu Rev Microbiol*, 22:519–548.

St-Pierre, F., Cui, L., Priest, D. G., Endy, D., Dodd, I. B., and Shearwin, K. E. (2013). One-Step Cloning and Chromosomal Integration of DNA. *ACS Synthetic Biology*, 2(9):537–541. PMID: 24050148.

Thomas, P. (2017). Making sense of snapshot data: ergodic principle for clonal cell populations. *J R Soc Interface*, 14(136).
