# Supplementary material for: Growth‐dependent heterogeneity in the DNA damage response in Escherichia coli
Source: Mol Syst Biol. 2022 May 27;18(5):e10441. doi: 10.15252/msb.202110441 (PMC9136515; doi:10.15252/msb.202110441)
Supplement: Supplementary file 2 — Expanded View Figures PDF [file MSB-18-e10441-s003.pdf]

Expanded View Figures

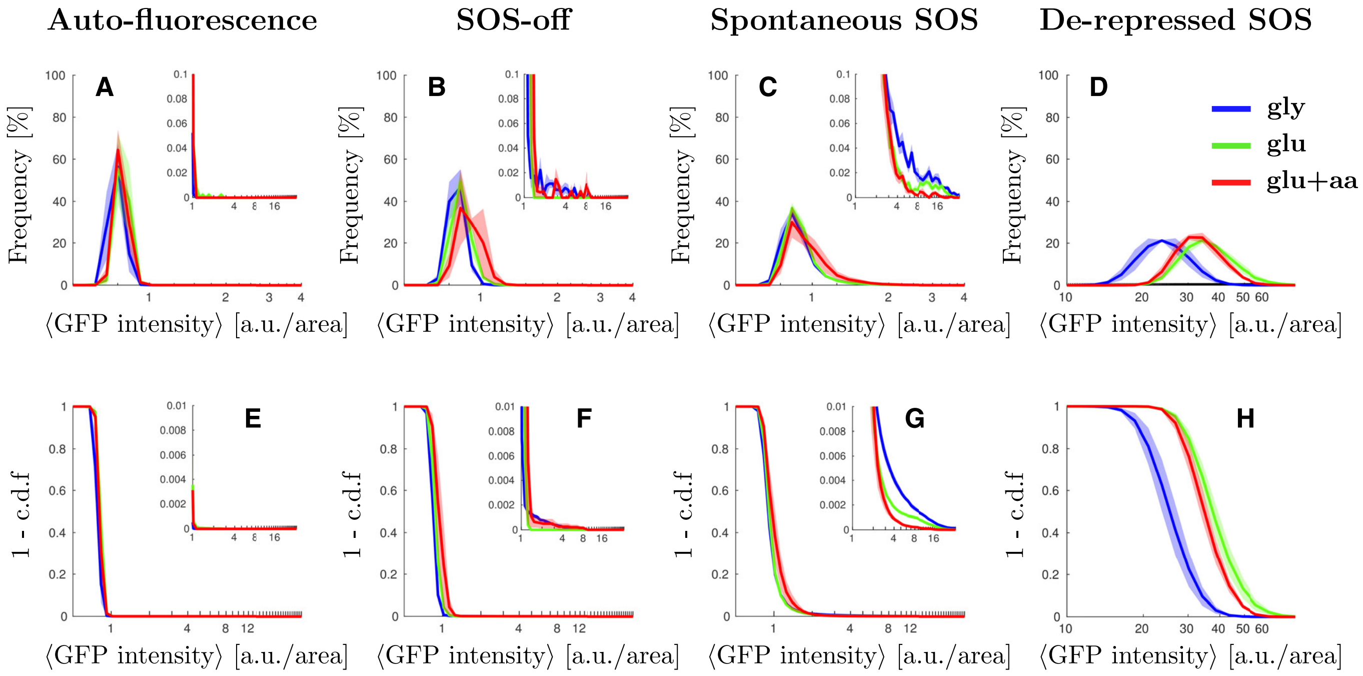

Figure EV1. Single cell distributions of GFP intensity without inducing DNA damage.

For all plots, growth conditions are: M9-glycerol (blue), M9-glucose (green), and M9-glucose+amino-acids (red). Solid lines represent the average and shaded area the standard error from three biological repeats. Inset: A magnification of the second peak at high SOS expression.

- A Steady-state distribution of GFP intensity from cell auto-fluorescence in different growth conditions.
- B Steady-state distribution of GFP intensity from SOS reporter *PsulA-mGFP* for cells unable to induce SOS (SOS-off, *lexA3* background) in different growth conditions. Figure panel reused from Fig 1B for comparison purposes.
- C Steady-state distribution of GFP intensity from SOS reporter *PsulA-mGFP* for wild type cells in different growth conditions. Figure panel reused from Fig 1A for comparison purposes.
- D Δ*lexA* Δ*sulA* background.
- E Steady-state cumulative distribution of GFP intensity from cell auto-fluorescence in different growth conditions.
- F Steady-state cumulative distribution of GFP intensity from SOS reporter *PsulA-mGFP* for cells unable to induce SOS (SOS-off, *lexA3* background) in different growth conditions.
- G Steady-state distribution of GFP intensity from SOS reporter *PsulA-mGFP* for wild type cells in different growth conditions.
- H Δ*lexA* Δ*sulA* background.

© 2022 The Authors

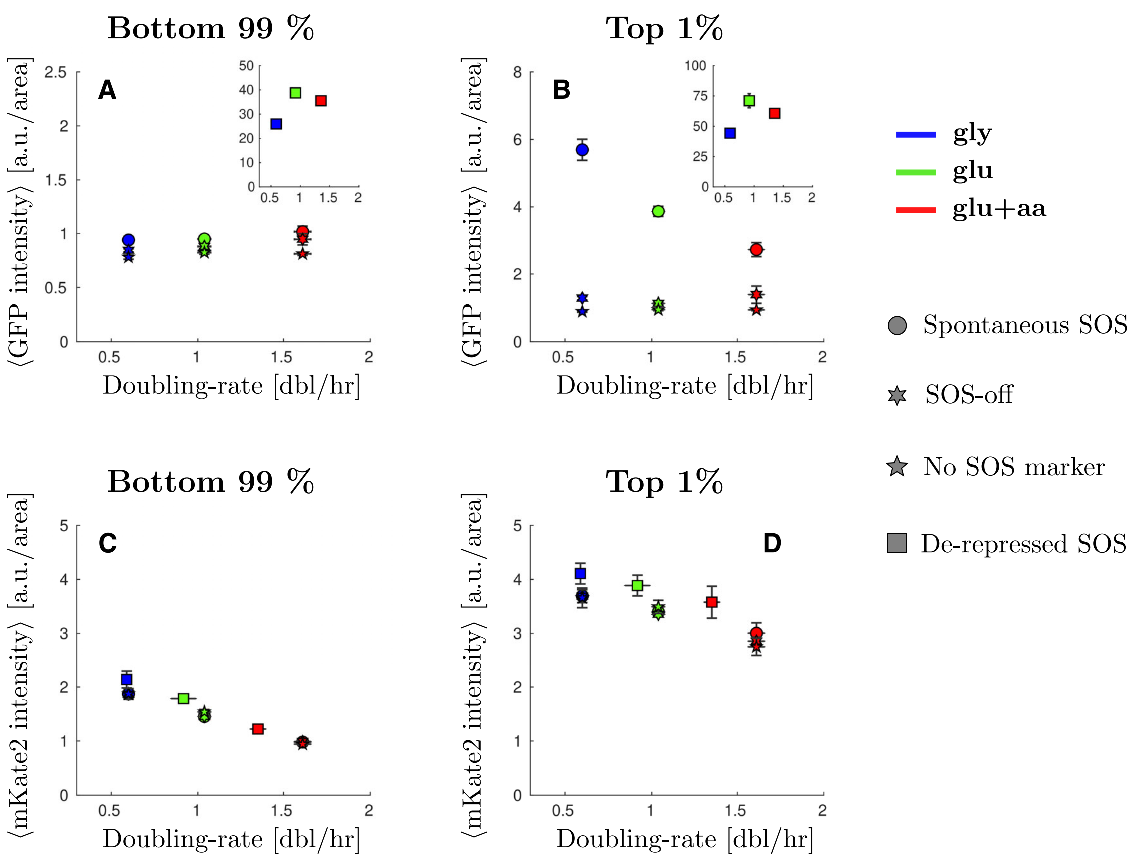

Figure EV2. Average GFP and mKate2 intensity without inducing DNA damage.

For all plots, growth conditions are: M9-glycerol (blue), M9-glucose (green), and M9-glucose+amino-acids (red). Points (dots WT strain, 6-points-stars SOS-off, *lexA3* background, 4-point-stars, autofluorescence) represent the average and bars the standard error from at least three biological repeats.

A Average GFP intensity from SOS reporter *PsulA-mGFP* for the bottom 99% of the population in different growth conditions. Inset: average GFP intensity in different growth conditions where SOS is de-repressed.

B Average GFP intensity from SOS reporter *PsulA-mGFP* for the top 1% of the population in different growth conditions. Inset: average GFP intensity in different growth conditions where SOS is de-repressed.

C Average mKate2 intensity from constitutive reporter *PtetO-mKate2* for the bottom 99% of the population in different growth conditions.

D Average mKate2 intensity from constitutive reporter *PtetO-mKate2* for the top 1% of the population in different growth conditions.

© 2022 The Authors

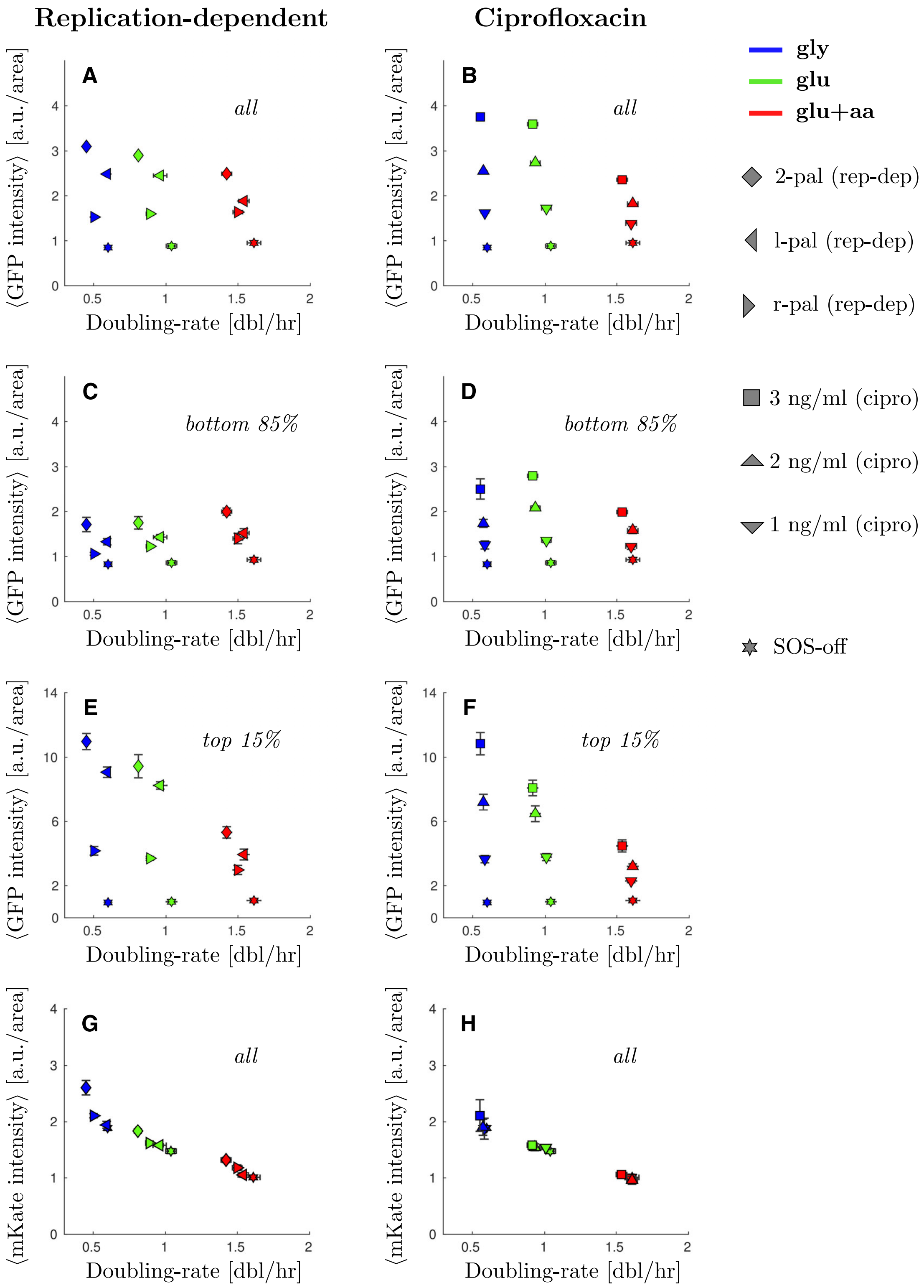
Replication-dependent
Ciprofloxacin
gly
glu
glu+aa
2-pal (rep-dep)
l-pal (rep-dep)
r-pal (rep-dep)
3 ng/ml (cipro)
2 ng/ml (cipro)
1 ng/ml (cipro)
SOS-off
A
B
C
D
E
F
G
H
all
bottom 85%
top 15%
⟨GFP intensity⟩ [a.u./area]
⟨mKate intensity⟩ [a.u./area]
Doubling-rate [dbl/hr]

Figure EV3.

Figure EV3. Average GFP and mKate2 intensity from inducing double-strand breaks.

For all plots, growth conditions are: M9-glycerol (blue), M9-glucose (green), and M9-glucose+amino-acids (red). Points represent the average and bars the standard error from at least three replicates done in different days.

A Average GFP intensity from SOS reporter *PsulA-mGFP* for the whole population under replication-dependent DSBs as a function of growth rate.
B Average GFP intensity from SOS reporter *PsulA-mGFP* for the whole population under ciprofloxacin as a function of growth rate.
C Average GFP intensity from SOS reporter *PsulA-mGFP* for the bottom 85% of the population under replication-dependent DSBs as a function of growth rate.
D Average GFP intensity from SOS reporter *PsulA-mGFP* for the bottom 85% of the population under ciprofloxacin as a function of growth rate.
E Average GFP intensity from SOS reporter *PsulA-mGFP* for the top 1% of the population under replication-dependent DSBs as a function of growth rate.
F Average GFP intensity from SOS reporter *PsulA-mGFP* for the top 1% of the population under ciprofloxacin as a function of growth rate.
G Average mKate2 intensity from constitutive reporter *PtetO-mKate2* for the whole population under replication-dependent DSBs as a function of growth rate.
H Average mKate2 intensity from constitutive reporter *PtetO-mKate2* for the whole population under ciprofloxacin as a function of growth rate.

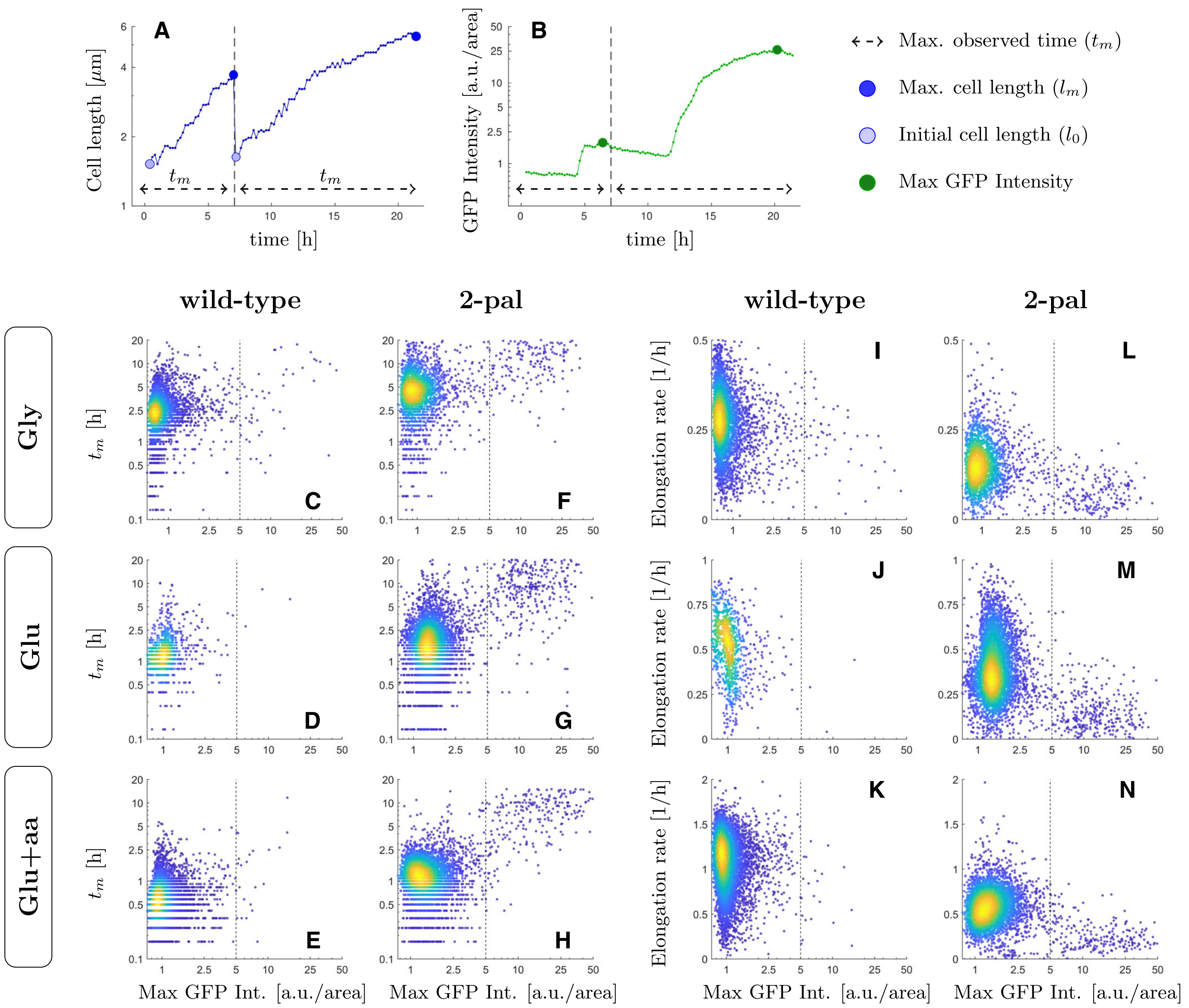

Figure EV4.

 © 2022 The Authors

Figure EV4. High-levels of SOS expression correlates with reduction in cell division and elongation rate under replication-dependent DNA-damage.

For every single-cell trajectory four metrics were computed as proxies for division and elongation rate reduction and its relation to GFP levels: the maximum observed time before a division or end of the tracking (t_m), the minimum and maximum cell lengths (l_O and l_m), and the maximum GFP intensity registered for that lineage.

A, B These metrics are illustrated for a lineage with two single-cell trajectories.
C–H The maximum observed time (t_m) was plotted against the maximum GFP intensity, comparing wild-type and cells undergoing replication-dependent DNA-damage (2-pal), where extreme values of GFP intensity correlate with significant delays in cell division only for the 2-pal strain.
I–N We plot a proxy for the cell elongation rate computed as $\log(l_m - l_O)/t_m$ against the maximum GFP intensity, where extreme values in GFP intensity correlate with a reduction in elongation rate as compared to the rest of the population.

Data information: Throughout panels C to N, colours represent the 2D density for each individual dataset, where yellow and blue denote high and low density, respectively. The vertical dashed line denotes the cutoff used throughout to discriminate high levels of SOS induction. Panels C-H contains data from 4,340, 1,016, 7,393, 2,572, 5,978, and 9,880 cell cycles, respectively.

© 2022 The Authors
