# Supplementary material for: Growth‐dependent heterogeneity in the DNA damage response in Escherichia coli
Source: Mol Syst Biol. 2022 May 27;18(5):e10441. doi: 10.15252/msb.202110441 (PMC9136515; doi:10.15252/msb.202110441)
Supplement: Supplementary file 5 [file MSB-18-e10441-s005.pdf]

molecular systems biology

Growth-dependent heterogeneity in the DNA damage response in Escherichia coli

Sebastián Jaramillo-Riveri, James Broughton, Alexander McVey, Teuta Pilizota, Matthew Scott, and Meriem El Karoui
DOI: 10.15252/msb.202110441

Corresponding author(s): Meriem El Karoui (meriem.elkaroui@ed.ac.uk)

Review Timeline:

Submission Date:	10th May 21
Editorial Decision:	9th Jun 21
Revision Received:	17th Feb 22
Editorial Decision:	17th Mar 22
Revision Received:	13th Apr 22
Accepted:	27th Apr 22

Editor: Maria Polychronidou

Transaction Report:

(Note: With the exception of the correction of typographical or spelling errors that could be a source of ambiguity, letters and reports are not edited. Depending on transfer agreements, referee reports obtained elsewhere may or may not be included in this compilation. Referee reports are anonymous unless the Referee chooses to sign their reports.)

Thank you again for submitting your work to Molecular Systems Biology. We have now heard back from the two referees who agreed to evaluate your study. Overall, the reviewers acknowledge that the presented findings seem potentially interesting. They raise however a series of concerns, which we would ask you to address in a revision.

I think that the recommendations of the reviewers are rather clear and therefore I do not see the need to repeat the points listed below. Several of the comments of reviewer #1 refer to the need to better support the main conclusions and they need to be carefully addressed. All other issues raised by the reviewers need to be satisfactorily addressed. Please contact me in case you would like to discuss in further detail any of the issues raised.

On a more editorial level, we would ask you to address the following points:

Reviewer #1:

In this work, Jaramillo-Riveri and colleagues investigate the DNA damage (SOS) response in E. coli at the single-cell level. They vary the nutrient composition of the growth medium, which leads to different steady state growth rates, and induce the SOS response by causing DNA double strand breaks in different ways. The first main finding is that the induction of the SOS response is highly heterogeneous with a larger fraction of cells strongly inducing this response in nutrient conditions that lead to slower growth. By observing cell growth and SOS induction using a GFP reporter in a "mother machine" microfluidic device, the authors find that this effect occurs despite the rate of SOS induction being lower at lower growth rates, as cells with low SOS induction grow faster.

Heterogeneity at the single-cell level and its coupling to growth is certainly a timely and interesting topic. Due to its detailed characterization in decades of work, the E. coli SOS response is an ideal model system to study this problem. The present work uses state-of-the-art techniques to tackle this problem at the single cell level. In particular, the single-cell assays in which the dynamics of cell growth and SOS induction are observed are a strength of this work. The main finding that a larger fraction of cells highly induce the SOS response in poorer nutrient environments is new and potentially interesting. However, it may be slightly exaggerated how 'surprising' or 'counter-intuitive' this result is and its biological relevance and implications do not become entirely clear. For many in the systems biology community, it will be relatively clear that different growth rates of subpopulations need to be taken into account to understand their abundances in the population at any given time point. Several key factors that underlie this phenomenon (heterogeneous SOS induction, inhibition of cell division by sulA) are known from previous work; the main new observations are the single-cell growth rates and the direct estimate of the SOS induction rate from single-cell assays. Overall, this work is potentially interesting for a broader audience but I have several major concerns about key technical aspects and the interpretation of the experimental data (detailed below) that would be critical to resolve.

Major points

1. The absolute fluorescence intensity of the GFP expressed from the SOS response promoter is an indirect reporter that is difficult to interpret at different growth rates. The main conclusions of this work strongly rely on these data. The GFP intensity in Fig 1E and Fig 2E,F seems to largely follow the expectation from bacterial growth laws for a stable protein expressed from a constitutive promoter (Scott, Science, 2010). The fact that no change in SOS induction is observed for the bottom 85% (Fig 2) or 99% (Fig 1) of the population does not rule out that this behavior is simply the general effect observed for most constitutive promoters because the lower GFP signals are close to the detection limit (autofluorescence, Suppl Fig 2), which may preclude detecting any changes. Importantly, this problem affects both the observed fraction of cells that highly induce the SOS response (Fig 1A and Fig 2A,B) and the estimate of the switching rate (Fig 3D). How do Fig 1A and Fig 2A,B and their interpretation change if GFP intensity on the x-axes is multiplied by the growth rate (to roughly correct for the general trend expected for a stable protein like GFP)? How is the analysis of the switching rate (Fig 3D), which relies on using the same GFP threshold in different growth conditions, affected by systematic changes in mean GFP level resulting from growth rate changes? It may be possible to correct for this general trend in different ways, but simply comparing absolute GFP intensities at different growth rates as currently done is doubtful and could lead to artifacts. Control experiments comparing the results for the SOS reporter to other (constitutive) promoters in the same assays could also help to strengthen this point.

2. Parts of the main text suggest that the distribution of SOS induction levels is bimodal, which would considerably facilitate the interpretation, but from the data shown that does not seem to be the case. Specifically, the distributions shown in Fig 1A and Fig 2A,B do not look bimodal. Hence, the distinction between high and low SOS states relies on an arbitrary threshold. However, the third paragraph on p. 7 suggests that there are two clearly distinct populations; this paragraph and other relevant parts of the text should be revised to clarify this point. Alternatively, stronger evidence for bimodality could be provided; for example, the distributions of growth rates and their correlation with SOS induction levels from the single-cell experiments (Suppl. Fig. 6 and 7) should be shown.

3. It is not clear if the observed higher switching rate in rich nutrient conditions is specific for the SOS response. A potential alternative explanation for this phenomenon is that such responses are generally faster in faster-growing cells (as many relaxation times of the system simply scale with the generation time). A helpful control experiment to validate that the effect is specific for the SOS response would be to use a common inducible promoter (e.g. P_lac) driving GFP, observe GFP trajectories as in Fig 3D upon inducer addition, and perform the same analysis as in Fig 3E.

4. Since quantitative growth rate differences are central to this work, it is a serious concern that the observed growth rates are lower in the microfluidic device. According to the top paragraph on p. 9, growth rate is lower by 20% in several of the growth media used, which is not negligible. This problem actually seems to be more severe than described in the main text: For the data shown in Fig 4D,E the batch culture growth rate in gly seems to be almost twice as high as in the mother machine, in glu+aa it seems about 50% higher. Indeed, this may be partly explained by filamentation causing an overestimation of growth rate measured by OD (p. 9, top). It would be helpful to validate if this is the case by CFU measurements (instead of OD) over time for the relevant growth conditions. If there are any remaining discrepancies, it will be important to explain in more detail why they are not problematic for the conclusions drawn.

5. The model in Fig 4 correctly predicts the trend of increasing fractions of cells with high SOS induction at lower growth rates and I agree that absolute quantitative agreement between model and experiment is unlikely (for the reasons explained in the

middle paragraph on p.9 and others). However, it is less clear why the relative increase is also drastically lower in the model: There is a ~3-fold increase from glu+aa to gly in the experiments (Fig 4E) but a predicted increase of only ~50% for the same comparison in the model prediction (Fig 4D). The origin of this discrepancy and its implications for the interpretation of the data need to be better explained.

Minor points

1. The slight increase of GFP signal at higher growth rate mentioned in the main text (p. 6, top paragraph) is hardly visible in Fig 2C (it is clearer in Fig 2A). The higher number of replication forks would suggest a much stronger increase here. Consider rephrasing the corresponding text on p. 6.

2. Correlations between the single-cell quantities observed in the microfluidic experiments in Fig 3 (division time, elongation rate, GFP intensity) should be analyzed and shown. These are important to corroborate the conclusions in the top paragraph on p. 8 (e.g. elongation rate should anti-correlate with SOS induction to support these conclusions).

Reviewer #2:

This paper reports on a single-cell analysis of the growth-rate dependence of the SOS response in E. coli. The authors determine distributions of SOS gene expression under different growth conditions and different sources of DNA damage to induce the SOS response. While the observe some overall effects on the whole population, the dominant effect is at the level of population heterogeneity. The observe a small subpopulation with high expression level. It is mostly that subpopulation that changes SOS expression between growth conditions. Time lapse microscopy and growth in a mother machine are then used to characterize the dynamics, specifically transitions between the two subpopulations.
An apparent contradiction that the rate of SOS induction is higher in rich medium, but that the fraction of SOS-high cells is lower, is explained with a mathematical model from earlier work on population heterogeneity.

This is an excellent work with interesting and convincing results, combining different experimental methods in a fruitful way and opening up a new view on the SOS response reflecting the interplay of gene expression and growth. Given the importance of that pathway, this approach can be expected to result in further important results. Overall, I am strongly in favor of accepting this paper and only have a few comments on some aspects.

1) I think some more quantification of how reproducible the small peak in the intensity histograms is might be helpful. This could also give a better idea of the variability in the high SOS population fraction.

2) The growth rate dependence of expression in the high SOS population is determined by the top 1% or 15 % of the population in terms of expression level. If the cutoff between the two subpopulation changes with growth rate, these results might be affected. In principle, a two-peak fit to the intensity histograms could give both the size of the subpopulations and their average intensities. At least a discussion of how the cutoff percentage was chosen and whether it affects the results would be helpful here.

3) Fig 2A,B: The main peak shifts, but for Ciprofloxacin, this seems to be not systematic. Are these medium-specific effects? Can growth rate still be considered as the dominant variable here?

4) How do the filamentous high SOS cells return to normal growth? If a filament divides multiple times and gives rise to multiple normal cells, the switching rate beta might play a role. Or is competition on growth still dominant?. Also, are filaments stably kept in the mother machine or are the carried away by the flow when they stick out of the trapping channels?

5) The general statement that slow growth conditions generically enrich subpopulations like the one studied here (last paragraph), is reasonably well known from persisters, where it is a consequence of exactly the equation for f2, which there expresses the persister fraction.

Point by point answers:
Reviewer 1

Overall, this work is potentially interesting for a broader audience but I have several major concerns about key technical aspects and the interpretation of the experimental data (detailed below) that would be critical to resolve.

The University of Edinburgh is a charitable body, registered in Scotland, with registration number SC005336.

We thank the reviewer for their interest in our work. We have addressed all the technical concerns below.
To make the reading of our new version easier, all major modifications in the manuscript are in blue.
Major points

1. The absolute fluorescence intensity of the GFP expressed from the SOS response promoter is an indirect reporter that is difficult to interpret at different growth rates.

The data that we provide is actually GFP fluorescence intensity ***per area*** (not absolute fluorescent levels) which is, therefore, a proxy for GFP concentration and allows comparisons at different growth rates. We use the term GFP intensity to refer to our calculation and have explained the calculation in the first paragraph of the results section. This is also explained in the legend of Figure 1.

We realized when reading the reviewer's comments that our initial text was very confusing because we referred to GFP levels in arbitrary fluorescence unit (a. u.) and not a. u./area (which is what we had calculated and were reporting). We apologize for this misleading mistake and have corrected it in the text. All the axis of the graphs referring to this data are now labelled a. u./area

The main conclusions of this work strongly rely on these data. The GFP intensity in Fig 1E and Fig 2E,F seems to largely follow the expectation from bacterial growth laws for a stable protein expressed from a constitutive promoter (Scott, Science, 2010).

We thank the reviewer for this comment which has helped us clarify the interpretation of our results. We agree that, indeed, a possible interpretation of the data provided in Figure 1E and Fig 2E, F is compatible with the following explanation: in cells where the SOS regulon is de-repressed, an SOS regulated protein behaves similarly to a constitutive stable protein and its concentration is therefore negatively correlated with growth rate (as shown in Scott, Science, 2010). We have added new data (Figure EV2, C, D; Figure EV3 G, H) to show that we indeed observe a negative correlation of constitutively expressed mKate with the growth rate, both with and without DNA damage.

However, if this were the only explanation, we would expect to observe a negative correlation between SOS expression and growth rate when we fully de-repress SOS as is the case in a Δ*lexA* mutant (note that we used a Δ*lexA* Δ*sulA* as a simple Δ*lexA is* not viable) . This is not what we observe: we have added new data in Figure EV 1D and insets of figure EV2 A, B which show that we observe a ***lower*** concentration of GFP driven by an SOS promoter at the low growth rate compared to the intermediate and fast growth rate. Therefore, our data suggest that the simple growth dependence predicted by the "bacterial growth laws" is not sufficient to explain our observations.

We have added an explanation and a reference to the additional figures in the third paragraph of the Results section "**The fraction of cells showing high levels of SOS expression induced by replication-dependent DSBs increases in slow-growth conditions**".

The fact that no change in SOS induction is observed for the bottom 85% (Fig 2) or 99% (Fig 1) of the population does not rule out that this behavior is simply the general effect observed for most constitutive promoters because the lower GFP signals are close to the detection limit (autofluorescence, Suppl Fig 2), which may preclude detecting any changes.

We think an alternative explanation is more likely than the one suggested by the reviewer for the following reasons. As explained above, GFP expression from a fully de-repressed SOS promoter (in which expression is very high and therefore not subjected to detection limits) does not show a growth dependence similar to a constitutive protein suggesting that the "bacterial growth laws" are not enough to fully explain our data.

The University of Edinburgh is a charitable body, registered in Scotland, with registration number SC005336.

However, for cells where induction of SOS is low, it is very likely that expression of GFP is still partially repressed by LexA: therefore GFP expression is subject to negative feedback because LexA negatively regulates its own expression. In this case, we do not expect any growth dependence of expression (Klummp *et al*. Cell, 2009 Dec 24;139(7):1366-75) and indeed this is what also we observe in the SOS-off situation where LexA cannot be cleaved (supplementary Figure EV1B, Figure EV2 A,B, 6 points stars).

Importantly, this problem affects both the observed fraction of cells that highly induce the SOS response (Fig 1A and Fig 2A,B) and the estimate of the switching rate (Fig 3D). How do Fig 1A and Fig 2A,B and their interpretation change if GFP intensity on the x-axes is multiplied by the growth rate (to roughly correct for the general trend expected for a stable protein like GFP)?

As explained above, the fully-derepressed expression (lexA-; Figure EV 1D and insets of figure EV 2A & 2B) precludes the interpretation that the negative correlation with growth rate shares the same origin as the negative correlation observed for constitutively expressed proteins.

How is the analysis of the switching rate (Fig 3D), which relies on using the same GFP threshold in different growth conditions, affected by systematic changes in mean GFP level resulting from growth rate changes?

As mentioned above our analysis is based on GFP concentration (a. u./area) and therefore already corrects for the different dilution rates associated with different growth rates. We have specifically chosen a high cut-off to define the population of High SOS cells so that the population estimates are not affected by growth conditions (see below with additional explanation based on figure EV4).

Moreover, if our correction does not fully account for growth rate-dependent expression changes, we would expect the contribution of growth rate to *increase* GFP concentration in slow growth condition (as per the bacterial growth laws) and therefore to potentially lead to *over-estimation* of the switching rate in slow growth compared to other growth condition. Despite this potential overestimation, we systematically observed a lower switching rate in the slow growth condition than in other conditions.

We are however aware that the choice of a GFP threshold is somewhat arbitrary which is why we provided an analysis of the estimate of the switching rate (alpha) as a function of the threshold, in Figure 3 E and appendix figure S6. We observe that the estimates of alpha are higher in fast growth conditions than in slow growth conditions over a large range of thresholds. For example, alpha at threshold 5 is **lower** in the slow growth condition than alpha at threshold 12 in the intermediate growth condition and alpha at threshold 20 in the fast growth condition (the maximum value that we could incorporate in our dataset given the GFP/area values we observe).
We are therefore confident that our alpha estimates are minimally affected by the threshold choices.

It may be possible to correct for this general trend in different ways, but simply comparing absolute GFP intensities at different growth rates as currently done is doubtful and could lead to artifacts.

As we explain above, we do not use absolute GFP intensities.

Control experiments comparing the results for the SOS reporter to other (constitutive) promoters in the same assays could also help to strengthen this point.

We have provided these data in Figure EV2(C, D) and figure EV3 (G, H). We observe the expected growth rate dependence for the mKate protein expressed from a constitutive reporter with higher mKate levels in slow growth conditions in keeping with expectations.

2. Parts of the main text suggest that the distribution of SOS induction levels is bimodal, which would considerably facilitate the interpretation, but from the data shown that does not seem to be the case. Specifically, the distributions shown in Fig 1A and Fig 2A,B do not look bimodal.

The referee is correct that the SOS distribution is not easily seen as bimodal in our figure. Following reviewer 2 suggestion, we have added two-peak fits to the distributions of SOS levels in the presence of the palindromes or ciprofloxacin in the appendix (appendix figure S8). The fits confirmed the presence of two populations with the average GFP intensity in the second population (varying between 2.27 to 3.5 a. u./area depending on growth conditions and type of DNA damage) being approximately twice that of first population. We chose to set a higher cut-off to define the second population at 5 a.u./area to exclude all the cells belonging to the first population (regardless of the growth conditions or type of DNA damage).

Hence, the distinction between high and low SOS states relies on an arbitrary threshold. However, the third paragraph on p. 7 suggests that there are two clearly distinct populations; this paragraph and other relevant parts of the text should be revised to clarify this point. Alternatively, stronger evidence for bimodality could be provided; for example, the distributions of growth rates and their correlation with SOS induction levels from the single-cell experiments (Suppl. Fig. 6 and 7) should be shown.

In figure EV4 we now provide the analysis suggested by the reviewer which confirms the presence of two different populations in the strain experiencing DNA damage, and thus provides additional support to the main hypotheses of our model.

We observe that in the presence of DNA damage, the main population shows relatively low levels of SOS induction (with GFP/area below 3 a.u./area) and normal elongation rates (ranging from 0.5 h^{-1} to 0.12 h^{-1} depending on the growth conditions). However, a second population of cells reach an SOS level of at least 5 a.u./area in all growth conditions (Figure EV4 F, G, H and L, M, N), which correlates with a much higher observation time (between 5 to up to 20 hours, corresponding to very low division rates or no division in the course of the experiment) and lower elongation rate of less than 0.1 h^{-1}. The fraction this second population corresponds to 7.89% in M9-gly, 4.58% in M9-glu and 3.4% in M9-glu+aa. This confirms the existence of two populations with markedly different behaviours with respect to SOS induction and division rates. In contrast, the WT strain had very few (between 0.43% in M9-gly and 0.014% in M9-glu-aa) outlier cells confirming that our observation is due to the presence of DNA damage.

We have added text in the first paragraph of the section "The transition rate to high-SOS state is higher in fast-growth conditions" to describe these data.

3. It is not clear if the observed higher switching rate in rich nutrient conditions is specific for the SOS response. A potential alternative explanation for this phenomenon is that such responses are generally faster in faster-growing cells (as many relaxation times of the system simply scale with the generation time). A helpful control experiment to validate that the effect is specific for the SOS response would be to use a common inducible promoter (e.g. P_lac) driving GFP, observe GFP trajectories as in Fig 3D upon inducer addition, and perform the same analysis as in Fig 3E.

Unfortunately, it is not possible to perform the experiment with pLac because the expression of the lactose permease is itself growth-dependent which would complicate the interpretation. However, we agree that our results might be more general and not specific to SOS. If the response rate to a stress is driven by the relaxation time of the system, and therefore the generation time, AND the stress affects division rates (e.g. after exposure to cell wall targeting antibiotics), then we would expect a behaviour similar to what we observe here, i.e. a higher proportion of highly-induced cells in slow growth conditions despite faster response time in fast

The University of Edinburgh is a charitable body, registered in Scotland, with registration number SC005336.

growth conditions. We have now mentioned this possibility in the second paragraph of the section "The transition rate to high-SOS state is higher in fast-growth conditions" and in the discussion.

4. Since quantitative growth rate differences are central to this work, it is a serious concern that the observed growth rates are lower in the microfluidic device. According to the top paragraph on p. 9, growth rate is lower by 20% in several of the growth media used, which is not negligible. This problem actually seems to be more severe than described in the main text: For the data shown in Fig 4D,E the batch culture growth rate in gly seems to be almost twice as high as in the mother machine, in glu+aa it seems about 50% higher.
Indeed, this may be partly explained by filamentation causing an overestimation of growth rate measured by OD (p. 9, top). It would be helpful to validate if this is the case by CFU measurements (instead of OD) over time for the relevant growth conditions. If there are any remaining discrepancies, it will be important to explain in more detail why they are not problematic for the conclusions drawn

To address the reviewer's concern on the description of the difference in the main text, we have reworded the explanation to make it clearer that the difference of 20% is observed for the WT strain and that it is more pronounced for the strain with two palindromes, including specifically mentioning the 50% difference for the strain carrying 2 palindromes.

To address the origin of the difference, and its effect on our data interpretation, we have performed the suggested experiments (presented in appendix table 1 and figure S7). Specifically, when we compare the mother machine doubling rate with CFU measurements, we observe a 9% difference for the strain carrying 2 palindromes in M9-glu-aa (the condition that had the highest discrepancy). The doubling rate from the CFU experiment is 1.08 dbl/h and 0.98 dbl/h in the mother machine experiment.

The result suggests that the growth rate obtained by OD measurements is over-estimated especially when cell filaments in agreement with what is known on the nature of OD measurements (Stevenson et al., 2016).

5. The model in Fig 4 correctly predicts the trend of increasing fractions of cells with high SOS induction at lower growth rates and I agree that absolute quantitative agreement between model and experiment is unlikely (for the reasons explained in the middle paragraph on p.9 and others). However, it is less clear why the relative increase is also drastically lower in the model: There is a ~3-fold increase from glu+aa to gly in the experiments (Fig 4E) but a predicted increase of only ~50% for the same comparison in the model prediction (Fig 4D). The origin of this discrepancy and its implications for the interpretation of the data need to be better explained.

The relative increase is underestimated in our prediction because we under-estimated the fraction of high SOS in low nutrient conditions. To correct for this under-estimation, we revised our model and used a more precise estimate of the faction of high SOS cells (f_2). In poor nutrients, the division rate of high SOS cell (λ_2) is probably not negligible as the low SOS cells are also dividing slowly. Therefore, a better estimate for $f_2$would be $f_2 \approx \frac{\alpha}{\lambda_1}\left(\frac{1}{1-\frac{\lambda_2}{\lambda_1}}\right)$ (Appendix equation 8). In the mother machine experiments, we did not observe enough cell divisions to accurately estimate $\lambda_2$in all growth conditions. However, data presented in Figure EV4 suggest a rough approximation for $\lambda_2 \approx 0.1$h^{-1}, based on which we can correct f_2. We obtain ≈15% for M9-gly ≈7.1% for M9-glu, values that are much closer to the fraction observed in batch experiments. We have added this explanation at the end of section "**A mathematical model based on two competing subpopulations explains the large fraction of high SOS cells observed in slow growth conditions".**

The University of Edinburgh is a charitable body, registered in Scotland, with registration number SC005336.

Minor points

1. The slight increase of GFP signal at higher growth rate mentioned in the main text (p. 6, top paragraph) is hardly visible in Fig 2C (it is clearer in Fig 2A). The higher number of replication forks would suggest a much stronger increase here. Consider rephrasing the corresponding text on p. 6.

Whilst the higher number of replication fork leads to a stronger increase of the number of DSBs, it also leads to a higher number of homologous regions that can be used for repair potentially limiting SOS induction. We have added a sentence to clarify this the paragraph **"The fraction of cells showing high levels of SOS expression induced by replication-dependent DSBs increases in slow-growth conditions"**

2. Correlations between the single-cell quantities observed in the microfluidic experiments in Fig 3 (division time, elongation rate, GFP intensity) should be analyzed and shown. These are important to corroborate the conclusions in the top paragraph on p. 8 (e.g. elongation rate should anti-correlate with SOS induction to support these conclusions).

We have provided the analysis requested in Figure EV4 and describe the results in the first paragraph of the **"The transition rate to high-SOS state is higher in fast-growth conditions"** section. The results confirm that high SOS level cells show a low elongation rate in all growth conditions.

Reviewer #2:

This is an excellent work with interesting and convincing results, combining different experimental methods in a fruitful way and opening up a new view on the SOS response reflecting the interplay of gene expression and growth. Given the importance of that pathway, this approach can be expected to result in further important results. Overall, I am strongly in favor of accepting this paper and only have a few comments on some aspects.

We thank the reviewer for their very supportive comments

1) I think some more quantification of how reproducible the small peak in the intensity histograms is might be helpful. This could also give a better idea of the variability in the high SOS population fraction.

We provide in all the figures the standard error of at least three replicates in shaded area. However, we recognize that the area is difficult to see because it is very narrow (i. e. in Figure 1A, B). We have added a plot with the individual repeats super-imposed on each other (appendix figure S9) to give better visual information about the reproducibility of the high SOS population fraction.

2) The growth rate dependence of expression in the high SOS population is determined by the top 1% or 15 % of the population in terms of expression level. If the cutoff between the two subpopulation changes with growth rate, these results might be affected. In principle, a two-peak fit to the intensity histograms could give both the size of the subpopulations and their average intensities. At least a discussion of how the cutoff percentage was chosen and whether it affects the results would be helpful here.

We have added two-peak fits to the distributions of SOS levels in the presence of the palindromes or ciprofloxacin in the appendix (appendix Figure S8). The fits confirmed the presence of two populations with the average GFP intensity in the high-SOS population (varying between 2.27 to 3.5 a. u./area depending on growth conditions and type of DNA damage) being approximately twice that of low-SOS population. We chose to set a higher cut-off to define the

high-SOS population at 5 a.u./area to exclude all the cells belonging to the low-SOS population (regardless of the growth conditions or type of DNA damage). Moreover, as shown in figure EV4 this cut-off correlates with high division time and lower elongation rates.

3) Fig 2A,B: The main peak shifts, but for Ciprofloxacin, this seems to be not systematic. Are these medium-specific effects? Can growth rate still be considered as the dominant variable here?

We agree that the shift of the main peak for Ciprofloxacin does not show growth dependence (stated in the main text, 1st paragraph of the section "**The fraction of cells showing high levels of SOS expression induced by exposure to ciprofloxacin increases in slow-growth conditions**").
We do not know the cause for the phenomenon, it is possible that the uptake (or efflux) of the drug is specifically affected in one of the growth media. Therefore we carried all the subsequent mother machine experiments using our genetic system inducing DNA damage to avoid this complication (we have added this explanation in the same paragraph).

4) How do the filamentous high SOS cells return to normal growth? If a filament divides multiple times and gives rise to multiple normal cells, the switching rate beta might play a role. Or is competition on growth still dominant?. Also, are filaments stably kept in the mother machine or are the carried away by the flow when they stick out of the trapping channels?

The filaments that we observe tend to divide by "budding" providing a single small cell at the extremity, in agreement with previous reports (Raghunathan S. et al., Mol Biol Cell. 2020 Dec 15;31(26):2920-2931). We have added a sentence to explain this in the second paragraph of the section "**The transition rate to high-SOS state is higher in fast-growth conditions**".
Filaments in the strain carrying two palindromes are relatively short and therefore very few are carried away from the mother machine during the course of our experiment.

5) The general statement that slow growth conditions generically enrich subpopulations like the one studied here (last paragraph), is reasonably well known from persisters, where it is a consequence of exactly the equation for f2, which there expresses the persister fraction.

This is true which is why we stated in the second paragraph of the section "The transition rate to high-SOS state is higher in fast-growth conditions" that our model was based on those used for the analysis of persisters and we also mention the analogy with persisters in the last paragraph of the discussion. We also note that our observations are probably applicable to cell wall damaging antibiotics that induce filamentation.

RE: MSB-2021-10441R, Growth-dependent heterogeneity in the DNA damage response in Escherichia coli

Thank you for sending us your revised manuscript. We have now heard back from the two reviewers who were asked to evaluate your revised study. As you will see below, they think that the study has improved as a result of the performed revisions. They do however list a few remaining concerns, which we would ask you to address in a revision.

We would also ask you to address some remaining editorial issues listed below:

Reviewer #1:

The authors have considerably improved the manuscript and convincingly addressed most points I had raised with additional experiments and explanations in the text.

There was a point of confusion I would like to clarify: when referring to the "absolute GFP fluorescence intensity", I did not mean that the normalization to cell volume (area) is missing but rather that the observed fluorescence intensity per GFP molecule can depend on changes in the intracellular milieu (e.g. pH and various metabolites are known to affect it), which could occur in the different growth media and under stress. Certain metabolites also affect GFP autofluorescence. Direct controls for this would still be valuable since this entire work relies on these data but it is certainly challenging to perform such controls at the single-cell level.

The new experiment in the ΔlexA ΔsulA background is a helpful addition and I agree with the authors that it shows that the growth laws alone cannot explain the observed expression changes. However, this observation is slightly confusing since the promoter is presumably only regulated by lexA and should consequently behave like a constitutive promoter in a ΔlexA background. More importantly, I cannot follow how this experiment addresses the concern that the observed lack of a correlation of GFP signal with growth rate (e.g. in Fig 1F) may be a detection issue due to low GFP signal. Even if the authors think that other explanations are more likely, clarifying this technical point would still be helpful: are the low fluorescence intensities sufficiently above background to detect changes of this magnitude?

Minor:

The data shown in Fig EV4 nicely supports the presence of two clearly distinct populations of cells. However, the data in appendix Fig S8 is not helpful in this respect: none of the distributions appear bimodal and no analysis or fitting will change this. In particular, the fact that fitting two Gaussians gives reasonable agreement with the data does not strengthen this point because this would be the case for many skewed (but unimodal) distributions. The two-Gaussian fits further seem quite poor in the tails of the distributions. These resemble heavy-tailed distributions, possibly following a power law. I would remove this analysis and the corresponding parts in the main text and focus on the more convincing data in Fig EV4 for this point.

It would be helpful to make clearer which promoters control GFP and mKate2 expression, respectively, in Figures EV2 and EV3 (and their legends).

A side note: The figures were not numbered or labeled at all in the file I got and separated from their legend, which made it unnecessarily hard to review this.

Reviewer #2:

The authors have done some additional work in the revision of their nice manuscript. In particular, they have added some clarifications on technical aspect and some new analysis. I find the new two-peak fits quite convincing and I also like the additional data from the growth measurement via CFU rather than OD. This side result also reports an interesting caveat on measuring growth in the presence of DNA damage.

I was already in favor of acceptance for the previous version of the manuscript and recommend it now. I only have one minor comment, which should be addressed before publication.

A minor comment:
Fig S8: Here the description of the grey lines as single-Gaussian fit is confusing. I believe these are the two Gaussians that are

obtained from the 2-Gaussian fit, not the results of fitting one Gaussian. Please clarify. Also there is a type „Guassian" twice in the figure caption.

Reviewer #1:

The authors have considerably improved the manuscript and convincingly addressed most points I had raised with additional experiments and explanations in the text.

There was a point of confusion I would like to clarify: when referring to the "absolute GFP fluorescence intensity", I did not mean that the normalization to cell volume (area) is missing but rather that the observed fluorescence intensity per GFP molecule can depend on changes in the intracellular milieu (e.g. pH and various metabolites are known to affect it), which could occur in the different growth media and under stress. Certain metabolites also affect GFP autofluorescence. Direct controls for this would still be valuable since this entire work relies on these data but it is certainly challenging to perform such controls at the single-cell level.

It is indeed possible that fluorescent protein intensity varies depending on growth media. However, we note that when we measure the expression of mCherry driven by a constitutive promoter we observe a growth dependence similar to what was observed using an enzymatic assay (using beta-galactosidase) which is not subjected to potential media-dependent change of activity (see Scott et. al, Science, 2010, Fig 2C) suggesting that growth media does not influence mCherry fluorescence (see Figure 1 for comparison below).

Figures for referees not available.

Figure 1. Comparison of an enzyme (left) and a fluorescent protein (right) expression at different growth rates. Left panel copied from Figure 2C of Scott et al., 2010, showing growth rate dependence of beta-galactosidase expressed from a constitutive promoter (pTetO1); empty circles of different colors correspond to different growth media (circles with numbers correspond to cell exposed to the protein synthesis inhibitor chloramphenicol and are not relevant to our comparison). Right panel: copied from Figure EV3 panel G of our manuscript, growth rate dependence of mCherry concentration.

It is possible that the fluorescence dependence on media would be specific to GFP (and not mCherry). In preliminary work leading to this manuscript, we obtained data measuring the expression of beta-galactosidase driven by an SOS regulated promoter (pLexA) with and without DNA damage (induced by mitomycin C). As shown in Figure 2 below, we observe the same growth dependence with GFP and beta-galactosidase further indicating that our observations are not due to a variation of fluorescence in different media. We did not include the beta-galactosidase data in the manuscript because they were obtained using a different type of DNA damaging agent, in a slightly different strain background and most importantly do not allow single cell analysis.

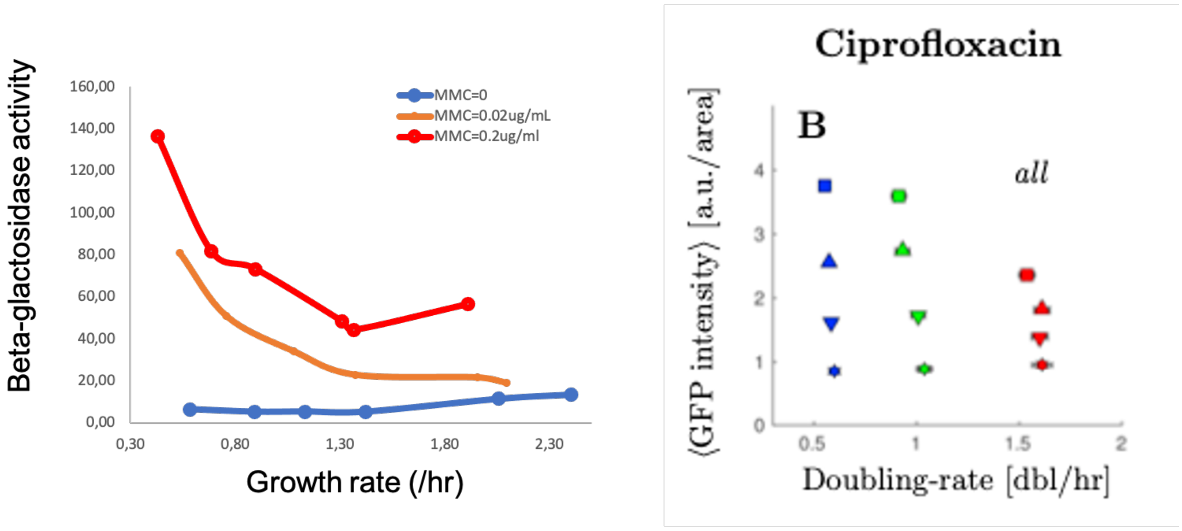

Figure 2: Comparison of an enzyme (left) and GFP expression driven by an SOS promoter at different growth rates. Left panel: our unpublished data showing population-based growth rate dependence of beta-galactosidase activity under exposure to mitomycin C (MMC) at 0.02 µg/ml (orange) and 0.2 µg/ml (red). In blue negative control without DNA damage. Right panel (from figure EV3 panel B): expression of GFP driven by an SOS promoter at different concentrations of ciprofloxacin (stars, no ciprofloxacin, triangle pointing down 1 ng/ml ciprofloxacin, triangle pointing up 2 ng/ml, square 3 ng/ml).

The new experiment in the ΔlexA ΔsulA background is a helpful addition and I agree with the authors that it shows that the growth laws alone cannot explain the observed expression changes. However, this observation is slightly confusing since the promoter is presumably only regulated by lexA and should consequently behave like a constitutive promoter in a ΔlexA background.

We agree with the referee that the observation is confusing because we would expect in first approximation a behavior similar to a constitutive promoter. Whilst we do not fully understand why we observe a different outcome, we think it might be linked to the much higher level of expression in this strain (more than 10 fold) compared to the DNA damage conditions we use. It is possible that the maximum level of SOS expression depends on growth conditions in a manner that is not simply explained by the growth laws but we feel this question is beyond the scope of this paper.

More importantly, I cannot follow how this experiment addresses the concern that the observed lack of a correlation of GFP signal with growth rate (e.g. in Fig 1F) may be a detection issue due to low GFP signal. Even if the authors think that other explanations are more likely, clarifying this technical point would still be helpful: are the low fluorescence intensities sufficiently above background to detect changes of this magnitude?

Figure EV2 panel C shows that we can detect growth dependence for the lower population (bottom 99%) of a constitutive promoter. The fact that we do not see it in Figure 1F is not surprising. In this panel, we present data for a mutant of LexA that cannot be cleaved after induction of DNA damage and therefore does not allow SOS induction. In this case, the promoter is fully repressed and subjected to negative feedback so no growth dependence is expected (see Klumpp et al. (2009) *Cell* **139**: 1366; Fig. 5A).

Minor:

The data shown in Fig EV4 nicely supports the presence of two clearly distinct populations of cells. However, the data in appendix Fig S8 is not helpful in this respect: none of the distributions appear bimodal and no analysis or fitting will change this. In particular, the fact that fitting two Gaussians

gives reasonable agreement with the data does not strengthen this point because this would be the case for many skewed (but unimodal) distributions. The two-Gaussian fits further seem quite poor in the tails of the distributions. These resemble heavy-tailed distributions, possibly following a power law. I would remove this analysis and the corresponding parts in the main text and focus on the more convincing data in Fig EV4 for this point.

We agree with the referee that Figure EV4 shows two distinct populations and importantly indicates that high SOS cells are clearly correlated with low division rates which strongly supports our model. The data provided in Figure S8 are indicative of bimodality but are less strong because we only have SOS induction level and not the division rates (as these data do not come from the mother machine experiments). We modified the text in the manuscript when describing the fits and now say that they are “indicative of potential” bimodality. We however feel that the fits should still appear in the manuscript as a supplementary figure (as suggested by referee 2).

It would be helpful to make clearer which promoters control GFP and mKate2 expression, respectively, in Figures EV2 and EV3 (and their legends).
We have modified the figure legend accordingly

A side note: The figures were not numbered or labeled at all in the file I got and separated from their legend, which made it unnecessarily hard to review this.
We apologize for this formatting issue

Reviewer #2:

The authors have done some additional work in the revision of their nice manuscript. In particular, they have added some clarifications on technical aspect and some new analysis. I find the new two-peak fits quite convincing and I also like the additional data from the growth measurement via CFU rather than OD. This side result also reports an interesting caveat on measuring growth in the presence of DNA damage.

I was already in favor of acceptance for the previous version of the manuscript and recommend it now. I only have one minor comment, which should be addressed before publication.

A minor comment:
Fig S8: Here the description of the grey lines as single-Gaussian fit is confusing. I believe these are the two Gaussians that are obtained from the 2-Gaussian fit, not the results of fitting one Gaussian. Please clarify. Also there is a type „Guassian" twice in the figure caption.

The figure legend was amended as requested.

ACCEPTED **27th Apr 2022**

Thank you again for sending us your revised manuscript. We are now satisfied with the modifications made and I am pleased to inform you that your paper has been accepted for publication.

EMBO Press Author Checklist

Corresponding Author Name: El Karoui	
Journal Submitted to: Molecular Systems Biology	
Manuscript Number: MSB-2021-10441	

USEFUL LINKS FOR COMPLETING THIS FORM

The EMBO Journal - Author Guidelines
EMBO Reports - Author Guidelines
Molecular Systems Biology - Author Guidelines
EMBO Molecular Medicine - Author Guidelines

Reporting Checklist for Life Science Articles (updated January 2022)

This checklist is adapted from Materials Design Analysis Reporting (MDAR) Checklist for Authors. MDAR establishes a minimum set of requirements in transparent reporting in the life sciences (see Statement of Task: 10.31222/osf.io/9sm4x). Please follow the journal's guidelines in preparing your manuscript.

Please note that a copy of this checklist will be published alongside your article.

Abridged guidelines for figures

1. Data

The data shown in figures should satisfy the following conditions:

- the data were obtained and processed according to the field's best practice and are presented to reflect the results of the experiments in an accurate and unbiased manner.
- ideally, figure panels should include only measurements that are directly comparable to each other and obtained with the same assay.
- plots include clearly labeled error bars for independent experiments and sample sizes. Unless justified, error bars should not be shown for technical replicates.
- if n<5, the individual data points from each experiment should be plotted. Any statistical test employed should be justified.
- Source Data should be included to report the data underlying figures according to the guidelines set out in the authorship guidelines on Data Presentation.

2. Captions

Each figure caption should contain the following information, for each panel where they are relevant:

- a specification of the experimental system investigated (eg cell line, species name).
- the assay(s) and method(s) used to carry out the reported observations and measurements.
- an explicit mention of the biological and chemical entity(ies) that are being measured.
- an explicit mention of the biological and chemical entity(ies) that are altered/varied/perturbed in a controlled manner.
- the exact sample size (n) for each experimental group/condition, given as a number, not a range;
- a description of the sample collection allowing the reader to understand whether the samples represent technical or biological replicates (including how many animals, litters, cultures, etc.).
- a statement of how many times the experiment shown was independently replicated in the laboratory.
- definitions of statistical methods and measures:
  - common tests, such as t-test (please specify whether paired vs. unpaired), simple χ2 tests, Wilcoxon and Mann-Whitney tests, can be unambiguously identified by name only, but more complex techniques should be described in the methods section;
  - are tests one-sided or two-sided?
  - are there adjustments for multiple comparisons?
  - exact statistical test results, e.g., P values = x but not P values < x;
  - definition of 'center values' as median or average;
  - definition of error bars as s.d. or s.e.m.

Please complete ALL of the questions below.
Select "Not Applicable" only when the requested information is not relevant for your study.

Materials

Newly Created Materials	Information included in the manuscript?	In which section is the information available? (Reagents and Tools Table, Materials and Methods, Figures, Data Availability Section)
New materials and reagents need to be available; do any restrictions apply?	Yes	No restrictions. Information in materials and methods and data availability section

Antibodies	Information included in the manuscript?	In which section is the information available? (Reagents and Tools Table, Materials and Methods, Figures, Data Availability Section)
For **antibodies** provide the following information:<br>- Commercial antibodies: RRID (if possible) or supplier name, catalogue number and or/clone number<br>- Non-commercial: RRID or citation	Not Applicable	

DNA and RNA sequences	Information included in the manuscript?	In which section is the information available? (Reagents and Tools Table, Materials and Methods, Figures, Data Availability Section)
**Short novel DNA or RNA including primers, probes**: provide the sequences.	Yes	Appendix table S5

Cell materials	Information included in the manuscript?	In which section is the information available? (Reagents and Tools Table, Materials and Methods, Figures, Data Availability Section)
**Cell lines:** Provide species information, strain. Provide accession number in repository **OR** supplier name, catalog number, clone number, and/**OR** RRID.	Yes	Apprendix table S1
**Primary cultures:** Provide species, strain, sex of origin, genetic modification status.	Not Applicable	
Report if the cell lines were recently **authenticated** (e.g., by STR profiling) and tested for mycoplasma contamination.	Not Applicable	

Experimental animals	Information included in the manuscript?	In which section is the information available? (Reagents and Tools Table, Materials and Methods, Figures, Data Availability Section)
**Laboratory animals or Model organisms:** Provide species, strain, sex, age, genetic modification status. Provide accession number in repository **OR** supplier name, catalog number, clone number, **OR** RRID.	Not Applicable	
**Animal observed in or captured from the field:** Provide species, sex, and age where possible.	Not Applicable	
Please detail **housing and husbandry conditions**.	Not Applicable	

Plants and microbes	Information included in the manuscript?	In which section is the information available? (Reagents and Tools Table, Materials and Methods, Figures, Data Availability Section)
**Plants:** provide species and strain, ecotype and cultivar where relevant, unique accession number if available, and source (including location for collected wild specimens).	Not Applicable	
**Microbes:** provide species and strain, unique accession number if available, and source.	Yes	Appendix table S2

Human research participants	Information included in the manuscript?	In which section is the information available? (Reagents and Tools Table, Materials and Methods, Figures, Data Availability Section)
If collected and within the bounds of privacy constraints report on age, sex and gender or ethnicity for all study participants.	Not Applicable	

Core facilities	Information included in the manuscript?	In which section is the information available? (Reagents and Tools Table, Materials and Methods, Figures, Data Availability Section)
If your work benefited from core facilities, was their service mentioned in the acknowledgments section?	Not Applicable	

Design

Study protocol	Information included in the manuscript?	In which section is the information available? (Reagents and Tools Table, Materials and Methods, Figures, Data Availability Section)
If study protocol has been **pre-registered, provide DOI in the manuscript**. For clinical trials, provide the trial registration number **OR** cite DOI.	Not Applicable	
Report the **clinical trial registration number** (at ClinicalTrials.gov or equivalent), where applicable.	Not Applicable	

Laboratory protocol	Information included in the manuscript?	In which section is the information available? (Reagents and Tools Table, Materials and Methods, Figures, Data Availability Section)
Provide DOI OR other citation details if **external detailed step-by-step protocols** are available.	Not Applicable	

Experimental study design and statistics	Information included in the manuscript?	In which section is the information available? (Reagents and Tools Table, Materials and Methods, Figures, Data Availability Section)

Include a statement about **sample size** estimate even if no statistical methods were used.	Yes	the sample size for each experiment is described in dataset EV1 and EV2
Were any steps taken to minimize the effects of subjective bias when allocating animals/samples to treatment (e.g. **randomization procedure**)? If yes, have they been described?	Not Applicable	
Include a statement about **blinding** even if no blinding was done.	Not Applicable	
Describe **inclusion/exclusion criteria** if samples or animals were excluded from the analysis. Were the criteria pre-established?<br><br>If sample or data points were omitted from analysis, report if this was due to attrition or intentional exclusion and provide justification.	Not Applicable	
For every figure, are **statistical tests** justified as appropriate? Do the data meet the assumptions of the tests (e.g., normal distribution)? Describe any methods used to assess it. Is there an estimate of variation within each group of data? Is the variance similar between the groups that are being statistically compared?	Yes	a detailed description of the modelling and statisical analysis is described in the appendix

Sample definition and in-laboratory replication	Information included in the manuscript?	In which section is the information available?<br>(Reagents and Tools Table, Materials and Methods, Figures, Data Availability Section)
In the figure legends: state number of times the experiment was **replicated** in laboratory.	Yes	figure legend
In the figure legends: define whether data describe **technical or biological replicates**.	Yes	figure legend

Ethics

Ethics	Information included in the manuscript?	In which section is the information available?<br>(Reagents and Tools Table, Materials and Methods, Figures, Data Availability Section)
Studies involving **human participants**: State details of **authority granting ethics approval** (IRB or equivalent committee(s), provide reference number for approval.	Not Applicable	
Studies involving **human participants**: Include a statement confirming that **informed consent** was obtained from all subjects and that the experiments conformed to the principles set out in the WMA Declaration of Helsinki and the Department of Health and Human Services Belmont Report.	Not Applicable	
Studies involving **human participants:** For publication of **patient photos**, include a statement confirming that consent to publish was obtained.	Not Applicable	
Studies involving experimental **animals**: State details of **authority granting ethics approval** (IRB or equivalent committee(s), provide reference number for approval. Include a statement of compliance with ethical regulations.	Not Applicable	
Studies involving **specimen and field samples:** State if relevant **permits** obtained, provide details of authority approving study; if none were required, explain why.	Not Applicable	

Dual Use Research of Concern (DURC)	Information included in the manuscript?	In which section is the information available?<br>(Reagents and Tools Table, Materials and Methods, Figures, Data Availability Section)
Could your study fall under dual use research restrictions? Please check biosecurity documents and list of **select agents and toxins** (CDC): https://www.selectagents.gov/sat/list.htm	Not Applicable	
If you used a select agent, is the security level of the lab appropriate and reported in the manuscript?	Not Applicable	
If a study is subject to dual use research of concern regulations, is the name of the **authority granting approval and reference number** for the regulatory approval provided in the manuscript?	Not Applicable	

Reporting

The MDAR framework recommends adoption of discipline-specific guidelines, established and endorsed through community initiatives. Journals have their own policy about requiring specific guidelines and recommendations to complement MDAR.

Adherence to community standards	Information included in the manuscript?	In which section is the information available?<br>(Reagents and Tools Table, Materials and Methods, Figures, Data Availability Section)
State if relevant guidelines or checklists (e.g., **ICMJE, MIBBI, ARRIVE, PRISMA**) have been followed or provided.	Not Applicable	
For **tumor marker prognostic studies,** we recommend that you follow the **REMARK** reporting guidelines (see link list at top right). See author guidelines, under 'Reporting Guidelines'. Please confirm you have followed these guidelines.	Not Applicable	
For **phase II and III randomized controlled trials**, please refer to the **CONSORT** flow diagram (see link list at top right) and submit the CONSORT checklist (see link list at top right) with your submission. See author guidelines, under 'Reporting Guidelines'. Please confirm you have submitted this list.	Not Applicable	

Data Availability

Data availability	Information included in the manuscript?	In which section is the information available?<br>(Reagents and Tools Table, Materials and Methods, Figures, Data Availability Section)
Have **primary datasets** been deposited according to the journal's guidelines (see 'Data Deposition' section) and the respective accession numbers provided in the Data Availability Section?	Yes	Data availabilty section
Were **human clinical and genomic datasets** deposited in a public access-controlled repository in accordance to ethical obligations to the patients and to the applicable consent agreement?	Not Applicable	
Are **computational models** that are central and integral to a study available without restrictions in a machine-readable form? Were the relevant accession numbers or links provided?	Yes	Data availabilty section
If publicly available data were reused, provide the respective **data citations in the reference list**.	Not Applicable	
